# Signatures of malignant cells and novel therapeutic targets revealed by single‐cell sequencing in lung adenocarcinoma

**DOI:** 10.1002/cam4.4547

**Published:** 2022-01-31

**Authors:** Jiaqi Liang, Zhencong Chen, Yiwei Huang, Guoshu Bi, Yunyi Bian, Xing Jin, Huan Zhang, Qihai Sui, Cheng Zhan, Qun Wang

**Affiliations:** ^1^ Department of Thoracic Surgery Zhongshan Hospital, Fudan University Shanghai China

**Keywords:** lung adenocarcinoma, single‐cell transcriptomics, therapeutic targets

## Abstract

**Background:**

Single‐cell transcriptomics has been used to investigate various tumors to elucidate the molecular distinction of all cell type compositions of a complex mix.

**Aims:**

This study aimed to investigate malignant‐cell‐specific genes to explore diagnostic and therapeutic biomarkers using single‐cell transcriptomic data of lung adenocarcinoma.

**Materials & Methods:**

10X single‐cell RNA‐seq data of fourteen patients with lung adenocarcinoma were analyzed. Genes that expressed differentially and those with higher confidence to distinguish tumor cells from normal cells were picked out using the ROC curves. The LASSO regression method was used to select most markedly correlated genes to predict the malignancy of every single cell within a model. We also conducted further experiments to determine their roles in lung cancer in vitro.

**Results:**

Twenty two thousand four hundred and ninety one tumor and 181 666 normal single cells were analyzed where 369 genes were found to be specifically expressed in single malignant cells. Seventy of them, encoding secreted or membrane‐bound proteins, showed involvement in cell‐to‐cell communications in tumor biology. KRT18 and the other six genes were identified as predictors to distinguish single malignant cells and were integrated to construct an accurate (96.1%) predicting model. Notably, IRX2, SPINK13, and CAPN8 outperformed the other four genes. Further experiments confirmed the upregulation of them in lung adenocarcinoma at both tissue and cell levels. Proliferative capacities of lung adenocarcinoma cells were attenuated by knocking‐down of either of them. However, targeting CAPN8, IRX2, or SPINK13 hardly exerted a cytotoxic effect on these cells.

**Discussion:**

Apart from the current model, similar tools were still warranted using single‐cell RNA‐seq data of more types of tumors. The three genes identified as potential therapeutic targets in the present study still need to be validated in more in lung cancer.

**Conclusion:**

Our model can aid the analyses of single‐cell sequencing data. CAPN8, IRX2, and SPINK13 may serve as novel targets of targeted and immune‐based therapies in lung adenocarcinoma.

## Graphical abstract

This study identified several tumor‐related genes that were scarcely revealed through analyses of bulk data provided with an effective approach to distinguish tumor cells from normal cells in single‐cell transcriptomic data. Several valuable genes used for promising targets in anticancer therapies in lung adenocarcinoma were discovered and validated in this work.

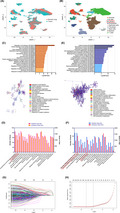

## 1 INTRODUCTION

Lung cancer is one of the most commonly diagnosed cancer and the leading cause of cancer death worldwide in the year 2020, with an estimated 2.2 million new cases and 1.8 million death, which represents more than one in ten (11.4%) cancers diagnosed and approximately one in five (18.0%) deaths.^1^ Traditionally, 85% of lung cancer is non‐small cell lung cancer (NSCLC), of which lung adenocarcinoma (LUAD) is the major histological subtype (66%).^2^, ^3^, ^4^

Yet, the presence of metastatic disease at the time of diagnosis in most patients poses clinical challenges, with a 5‐year overall survival of less than 30%.^5^, ^6^, ^7^, ^8^ Standard management of advanced patients now comprises surgery, chemoradiotherapy, various target therapies, and immunotherapies, which need to be greatly improved to manage survival effectively. Therefore, it is necessary to identify new malignant‐cell‐specific gene alterations and immune‐associated markers to expand the population suitable for targeted or immune‐based therapies.

Transcriptome sequencing has been a powerful technique to seek new therapeutic targets.^9^ Conventional bulk RNA‐sequencing (RNA‐seq) is one of the most used methods to delineate a transcriptome profile, but this cannot explore the molecular complexity and diversity of cells in a sample. While single‐cell transcriptomic analysis compensates for understanding the tumor microenvironment (TME),^10^, ^11^, ^12^ distinguishing malignant cells from normal cells in the TME for further independent studies is still challenging.

This study aimed to identify genes specifically expressed in single malignant cells by examining malignant and other cell types using single‐cell RNA sequencing (scRNA‐seq) data of LUAD. Our goal was to find those malignant‐cell‐specific genes with the potential to be therapeutic targets. Furthermore, we exploited a gene prediction model with a score to identify single malignant cells better with scRNA‐seq data. This model would lead to a new method beyond the limited applicability of traditional ways to separate tumor cells to be studied independently. Meanwhile, several novel biomarkers and their roles were spotted and validated in our work. We hope our results will promote the development of targeted and immune‐based therapies to herald the era of personalized medicine for LUAD patients.

## 2 SUBJECTS AND METHODS

### 2.1 Study cohorts and specimens

As previously reported, scRNA‐seq data of 14 LUAD patients under surgery in our department and two other independent cohorts of the ArraryExpress [Accession No. E‐MTAB‐6149 & E‐MTAB‐6653] and the Human Cell Atlas Data Coordination Platform [Accession No. PRJEB31843] were included.^13^, ^14^, ^15^ Tissue specimens for immunochemistry (IHC) were obtained from another 20 primary patients who received surgery during March 2021 and were histologically diagnosed with lung adenocarcinoma. Five of these patients were clinically diagnosed with advanced stages and the others with early‐stage tumors. Detailed information is available in the Table S1.

### 2.2 10x scRNA‐seq data analysis

Data were processed by R software (Version 3.6.1) as reported before.^13^ Briefly, cells with gene expression fewer than 200 or more than 7000 and those with more than 20% of mitochondrial genes were removed in the step of quality control first. Data were then normalized with a dimension reduction after scaling and centering features. “Harmony” R package was used to integrate data from different cohorts.^16^ Uniform manifold approximation and projection (UMAP) analysis were subsequently conducted.

The SingleR R package, the CellMarker dataset, and previous studies were combined for cell annotations in our analysis.^17^, ^18^, ^19^ The “FindAllMarkers” function was used to identify genes that expressed differentially (differentially expressed genes, DEG) between malignant cells and normal cells (False discovery rate [FDR] < 0.01 & |log2FC| ≥ 1). Trajectory analyses were performed using the monocle2 to explore the tumor‐reprogramming processes in the single cells.^20^

### 2.3 Construction of the prediction model to discriminate malignant cells

To detect a subset of genes with higher confidence to distinguish tumor cells in scRNA‐seq data, the area under the receiver operating characteristic (ROC) curve (AUC) was calculated on each of the DEGs using the pROC R package.^21^ Genes with a value of AUC more than 0.60 were considered acceptable for tumor‐cell discrimination and were pooled into the prediction gene set, in which genes encoding secreted or membrane‐bound proteins were selected using the subcellular localization data from the Human Protein Atlas (http://www.proteinatlas.org/).^22^

Gene Ontology (GO) and Kyoto Encyclopedia of Genes and Genomes (KEGG) pathway enrichment analyses were then conducted using the MetaScape (http://metascape.org/) (adjusted *p*‐value <0.01 and the number of enriched genes >3).^23^ Furthermore, diseases significantly associated with these genes were explored by the DisGeNET,^24^ a comprehensive dataset, together with the ToppGene platform (http://toppgene.cchmc.org).^25^ Statistic outputs were finally visualized using GraphPad Prism 8.0.

In Cox's proportional hazard model, all genes in the prediction gene set were screened by the least absolute shrinkage and selection operator (LASSO).^26^ Ten‐fold cross‐validation was adopted using the R package glmnet to determine eligible genes incorporated into the final prediction model. Appropriate parameter λ, which can present an optimally minimized residual sum of squares in the Cox's model, was selected. Final genes were pooled into a binomial logistic regression to construct an effective model to distinguish tumor cells.^26^, ^27^

### 2.4 Immunochemistry (IHC)

IHC was conducted to investigate further the expression disparity of certain genes within LUAD tissue specimens. New tissues were fixed at 4% PFA Fix Solution for 2 h, then sliced into 5‐μm sections after being embedded with paraffin. The slides were dewaxed, rehydrated, and stained with H&E or the GTvision™ HRP‐polymer anti‐mouse/rabbit IHC Kit (GeneTech). Rabbit anti‐IRX2 antibody (Dilution 1:50, #abs130413, Absin), rabbit anti‐SPINK13 antibody (Dilution 1:200, # ab122339, Abcam), and rabbit anti‐CAPN8 antibody (Dilution 1:10, #abs101783, Absin) were used, and equivalent phosphate‐buffered saline (PBS) was used as a negative control for primary antibodies. The slides were scanned at 100× and 400× using the Olympus BX51 microscope.

Images were judged by two researchers independently using the immunoreactivity score (IRS) [IRS = Staining intensity × Proportion of positive‐stained cells] as reported elsewhere.^28^ The staining intensity of colored cells was assessed as follows: 0 (colorless/negative), 1 (light yellow/weak), 2 (dark yellow/moderate), and 3 (yellowish‐brown/strong). The proportion score of positive‐stained cells was identified by the followings: 0 (0%), 1 (1–30%), 2 (31–60%), and 3 (61–100%). Gene was finally labeled “high‐expressed” with an IRS score of no less than 4. Otherwise, it was defined as “low‐expressed” in regions on the slides.

### 2.5 Cell culture and small interfering RNA (siRNA) transfection

Two RAS‐driven LUAD cell lines (A549 and NCI‐H1299) were purchased from the Chinese Academy of Science Cell Bank (https://www.cellbank.org.cn/). The cell lines were authenticated by short tandem repeat (STR) profiling before use. Cells were cultured in the DMEM (High Glucose), supplemented with 10% fetal calf serum (Every Green, Zhejiang Tianhang Biotechnology), as well as penicillin/streptomycin/amphotericin B (Beyotime Biotechnology). Cells were cultured in a humidified atmosphere of 95% air and 5% CO_2_ at 37°C.

They were transfected by lentivirus containing green fluorescent protein (GFP), and untransfected cells were filtered out by flow cytometry. Then GFP‐included cells were transfected with siRNAs at a 100 nM final concentration using the Lipofectamine8000 (Beyotime) and the Opti‐MEM (Thermo Fisher Scientific). siRNAs were designed by and purchased from the Guangzhou RiboBio Co, China. We used two different siRNAs separately targeting each gene (IRX2, SPINK13, and CAPN8) and two different siRNAs without targeting the human genome as controls. The targeting sequences of each siRNA can be accessed in the Table S2. The efficiency was assessed using quantitative real‐time polymerase chain reaction (qRT‐PCR) and western blot analysis after transfection. Primers used can be obtained in the Table S3.

### 2.6 Cell proliferation and apoptosis analysis

A total of 1500 cells in the logarithmic growth phase were digested and inoculated in black 96‐well plates (Life Science) for proliferation analysis. After 24, 48, 72, 96, and 120 h, cells in each well were photographed, and cell proliferation was measured according to corresponding fluorescence intensity using a Celigo cytometer (Cyntellect Inc.).

Cells were stained with APC‐conjugated Annexin V according to the manufacture's instruction (Annexin V Apoptosis Detection Kit APC, eBioScience, Cat. No. 88–8007), and Annexin V^+^ populations were analyzed by flow cytometry with a FACS AriaIII (BD Biosciences).

### 2.7 qRT‐PCR

Total RNAs of cells were extracted using TRNzol Universal (Tiangen Biotech) according to the manufacturer's guidelines. Then cDNA synthesis was performed using Hifair^®^ III 1st Strand cDNA Synthesis SuperMix for qPCR (gDNA Digester Plus) (Yeasen Biotechnology). Then qRT‐PCR was performed with QuantStudio5 (Thermo Fisher Scientific) using Hieff^®^ qPCR SYBR Green Master Mix (Low Rox Plus) (Yeasen Biotechnology) based on the manufacturer's guidelines. We used GAPDH as the endogenous control and the 2^–ΔΔCT^ method to calculate the gene expression.

### 2.8 Western blot

The siRNA‐transfected cells were treated using RIPA Lysis Buffer (Beyotime) with a proteinase inhibitor cocktail (TargetMol). Protein concentration was assessed using Enhanced BCA Protein Assay Kit (Beyotime). The same amounts (20 μg) of total proteins were separated using gel electrophoresis and then transferred to polyvinylidene fluoride membranes (Beyotime). After being incubated in milk for 2 h, the blots were incubated with Rabbit anti‐IRX2 antibody (Dilution 1:1000, #abs130413, Absin), rabbit anti‐SPINK13 antibody (Dilution 1:1000, #HPA036456, Atlas Antibodies AB), Rabbit anti‐CAPN8 antibody (Dilution 1:1000, #abs101783, Absin), overnight at 4°C, respectively. Meanwhile, mouse anti‐GAPDH antibody (Dilution 1:3000, #AA128, Beyotime) was used as endogenous control. After washing, blots were incubated with HRP‐conjugated secondary antibodies (Dilution 1:3000) for 2 h and visualized using BeyoECL Moon Kit (Beyotime).

## 3 RESULTS

### 3.1 Single‐cell transcriptomic profiling of LUAD patient tumor tissue identifies malignant and non‐malignant cells

Twenty‐nine samples of 26 LUAD patients (12 from FDZSH and 14 from the ArrayExpress), including 11 early and six advanced LUAD, together with 12 normal lung tissue samples, were included in the present study. Demographic and clinicopathological characteristics of all patients had been reported before.^13^ Sequencing data derived from cells from the two databases were mixed using the Harmony R package into a well‐integrated scRNA‐seq dataset. Finally, 204,157 cells were selected to generate an atlas after quality control.

**FIGURE 1:**
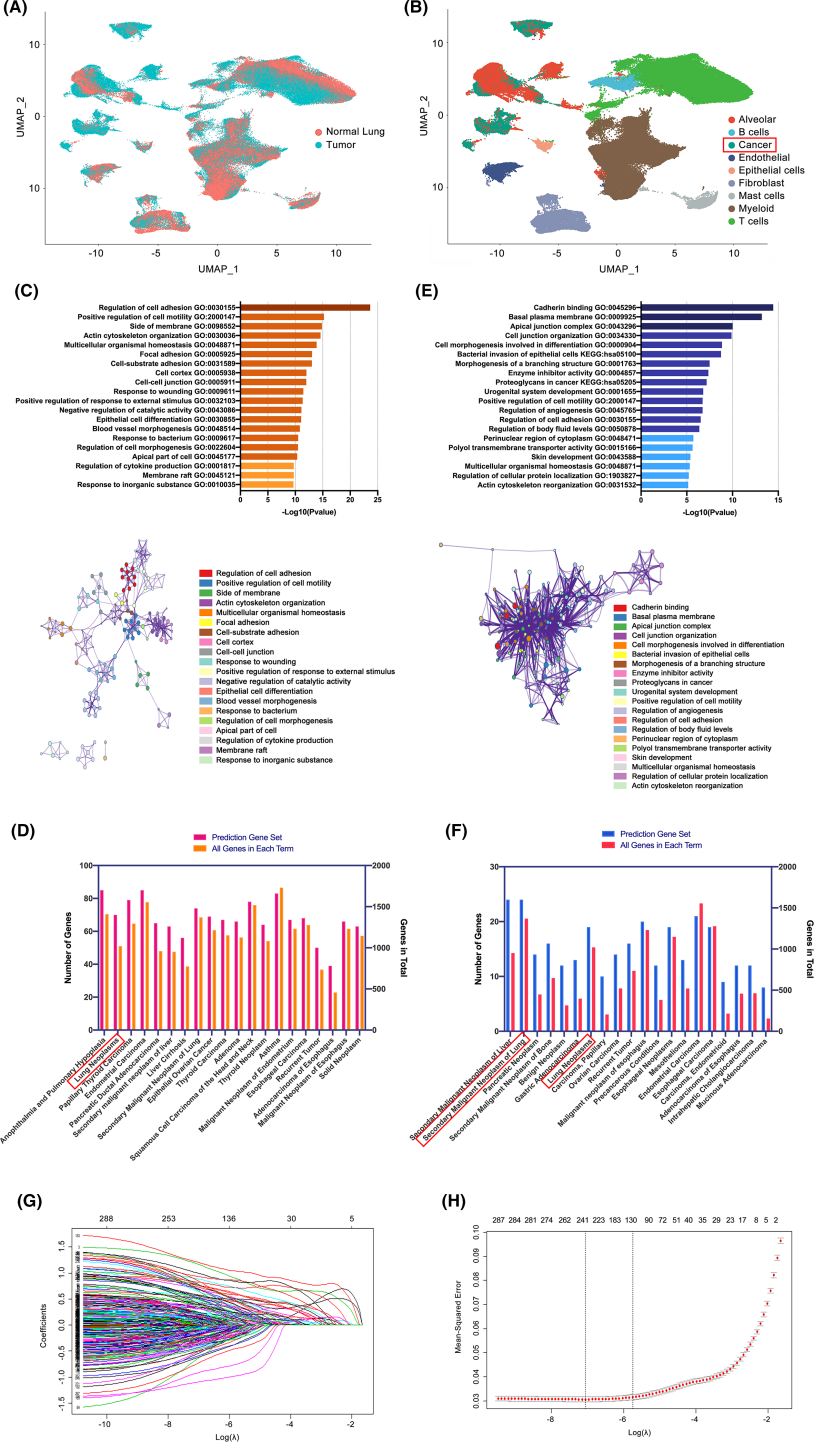
Single‐cell transcriptomic atlas of 29 samples from normal lung, early LUAD, and advanced LUAD tissues and results during the construction of the prediction model. (A) Feature (UMAP) plots to show the origins of single cells. (B) Cell type clusters of eligible single cells based on the expression of canonical marker genes. (C) Top 20 and network of enriched terms resulting from pathway and process enrichment analysis for candidates in the prediction gene set. Analyses were performed with sources of KEGG pathway and GO biological processes. Genes in the prediction gene set performed well (AUC higher than 0.60) in distinguishing single malignant cells. (D) Top 20 enriched human diseases for candidates in the prediction gene set. Analyses were performed using collections of genes involved in human diseases on the DisGeNET platform. (E) Top 20 and network of enriched terms resulting from pathway and process enrichment analysis for 70 genes encoding secreted or membrane‐bound proteins. (F) Top 20 enriched human diseases for 70 genes encoding secreted or membrane‐bound proteins. (G) Plots for LASSO cox regression coefficients over optimal penalty parameter values with all 369 genes in the prediction gene set. (H) Cross‐validation plot for penalty term in the LASSO cox regression model

Subsequently, dimensionality reduction and unsupervised clustering analysis were conducted, and nine cell clusters were identified, as we previously reported.^13^ As shown in Figure 1A,B, nine distinct cell lineages were cataloged. Six types—B cells, T cells, myeloid cells, fibroblasts, endothelial cells, and mast cells—were first identified primarily using the SingleR package and canonical markers in the CellMarker dataset.

The other three types—cancer cells, epithelial cells, and alveolar cells were identified and validated by three different methods. Cancer cells were determined by known markers, EPCAM and SOX4, while epithelial cells were identified by CAPS and TPPP3 and alveolar cells by SFTPC and SFTPA.^13^, ^14^, ^15^ Those determined cancer cells were, on the one hand, confirmed to be malignant, resulting from significantly higher copy number variations (CNVs) compared to reference cells using the “inferCNV”.^13^ Besides, cells in the cancer cell cluster were all confirmed to be originated from LUAD samples, whereas epithelial and alveolar cells were from normal lung tissues. Using the “scPred” R package, the identification results of malignant cells were validated to be robust since most of the identified cancer cells were not designated to either epithelial or alveolar clusters, on the other hand.

Thus, the three approaches concordantly segregated cells into malignant and normal subsets, ending up 22,491 tumor cells and 181,666 normal cells to proceed with our following research. Additional information in detail regarding cell clustering can be obtained from our previous work.^13^

### 3.2 Construction of model and a score for malignant cell discrimination with seven genes

In the sequencing profiles, we first identified 1655 DEGs between the tumor and normal cells in the current study, with the gene expression fold change (FC) no less than 1.41 or no more than 0.70 (|log2FC| ≥ 0.5). A total of 949 genes showed an elevation in tumor cells compared to normal ones, whereas the others showed a decrease.

Next, ROC curve analysis was adopted to assess the discriminating ability between malignant and normal cells of each DEG at the single‐cell level. Three hundred and sixty‐nine of all DEGs performed well (AUC higher than 0.60) in distinguishing between the two cell clusters (Table S4). Among them, 293 were upregulated and 76 were downregulated in tumor cells compared to the others.

These 369 genes were subsequently curated in a new prediction gene set. Consistently, they were found to be widely expressed in various types of tumors and significantly associated with biological functions, such as cell adhesion and motility regulation, resulting from the DisGeNET and GO&KEGG analyses as presented in Figure 1C,D.

Besides, we singled out 70 genes encoding secreted or membrane‐bound proteins, such as MUC1, CLDN4, referring to the subcellular location of each protein presented in The Human Protein Atlas (Table S4). Notably, these genes were demonstrated to be significantly related to secondary or recurrent tumors, and they were enriched more in cell‐to‐cell communications (Figure 1E,F). Genes in the prediction gene set can be obtained in the Table S4.

Using the LASSO method, all 369 genes in the prediction gene set were run to determine which can be used to screen malignant cells at a single‐cell level in combination. Figure 1G showed results on the 369 variables included in the LASSO regression and their corresponding coefficients for the different values of the penalty parameter. We observed that at a λ = 0.00002, all 369 variables remain in the model. As the penalty term λ increased, variables that remained longer in the model were discarded when others remained because they approached zero more quickly.

**FIGURE 2:**
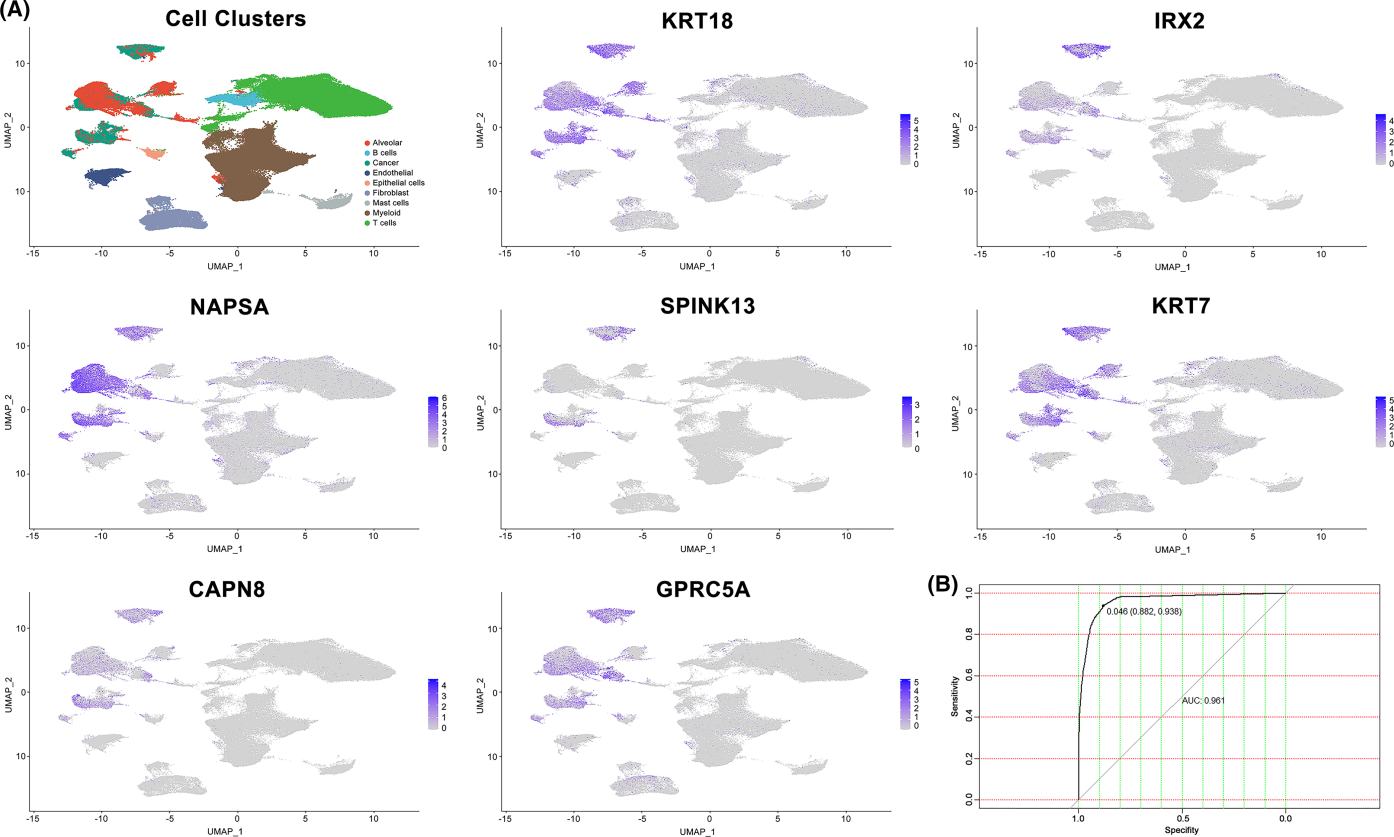
Expression level of the seven genes included in the model and ROC curve of the final model. (A) Expression of genes (KRT18, IRX2, NAPSA, SPINK13, KRT7, CAPN8, and GPRC5A) in every single‐cell type cluster. (B) ROC curve of the final prediction model. Results showed AUC was 0.961 with a sensitivity of 88.2% and a specificity of 93.8%

Specifically, as λ approached 0.1091, seven predictors—KRT18, IRX2, NAPSA, SPINK13, KRT7, CAPN8, and GPRC5A—conferred the largest signal in the model (Figure 1G,H). All of them showed an upregulation in our previously identified malignant cells compared to the non‐malignant cells (Table S4). AUCs of these seven genes ranged from 0.725 to 0.927, as depicted in Table 1. Their capability to distinguish malignant cells from others was demonstrated to be reliable, as shown in the gene feature maps visualized using UMAP (Figure 2A). Notably, among the seven genes, IRX2, SPINK13, CAPN8, and GPRC5A showed great superiority over their counterparts. They were expressed at low levels in definitive alveolar cells, which has often been the most difficult to be distinguished because the alveolar cells can express many of the same epithelial markers as cancer cells.

**TABLE 1:** Information of top 20 genes ranked according to AUC for single malignant cell distinguishing and genes included in the prediction model

Rank	Gene symbol	AUC	Sensitivity	Specificity	|log2FC|	Secreted or membrane‐bound proteins	Included in the regression model	Coefficients
1	KRT18	0.927	0.903	0.865	1.855	No	Yes	0.8449
2	KRT8	0.924	0.839	0.923	1.851	No	No	–
3	NAPSA	0.921	0.899	0.881	1.994	No	Yes	1.2584
4	MUC1	0.916	0.907	0.861	1.729	Yes	No	–
5	WFDC2	0.912	0.897	0.850	1.898	No	No	–
6	SFTPB	0.901	0.888	0.854	1.989	No	No	–
7	HOPX	0.900	0.879	0.827	1.693	No	No	–
8	KRT7	0.892	0.867	0.865	1.468	No	Yes	1.5488
9	EPCAM	0.885	0.885	0.837	1.485	Yes	No	–
10	KRT19	0.884	0.839	0.867	1.413	No	No	–
11	SFTA2	0.878	0.840	0.878	1.218	No	No	–
12	ZFP36L1	0.877	0.812	0.828	1.232	No	No	–
13	ERRFI1	0.874	0.842	0.831	1.671	No	No	–
14	C8orf4	0.873	0.840	0.833	2.120	No	No	–
15	ELF3	0.872	0.878	0.827	1.561	No	No	–
16	NKX2‐1	0.865	0.924	0.790	1.109	Yes	No	–
17	RNASE1	0.865	0.830	0.824	1.169	No	No	–
18	GPRC5A	0.864	0.813	0.781	1.025	Yes	Yes	0.6344
19	CLDN4	0.862	0.879	0.807	1.247	Yes	No	–
20	SLC34A2	0.861	0.875	0.822	1.461	Yes	No	–
…								
34	IRX2	0.835	0.950	0.701	1.734	No	Yes	0.7221
…								
64	CAPN8	0.806	0.949	0.652	0.942	No	Yes	0.4969
…						No		
215	SPINK13	0.725	0.992	0.456	1.010	Yes	Yes	2.3251

Abbreviations: AUC, area under the curve; FC, fold change.

A model consisting of the seven genes was developed using binomial logistic regression analysis. This model could directly achieve the likelihood of every single cell being judged to be malignant. It was defined as “single‐cell malignant score (scMS)” that was calculated with the expression of the seven selected genes as parameters in an equation: scMS = 1 + 1/(1 + e^−x^) = where x = −4.7082 + 0.8449 (KRT18) + 0.7221 (IRX2) + 1.2584 (NAPSA) + 2.3251 (SPINK13) + 1.5488 (KRT7) + 0.4969 (CAPN8) + 0.6344 (GPRC5A). Gene abundance should be scaled and centered after being acquired from the FPKM matrix representing normalized gene expression count data. The optimal cut‐point for evaluating malignant cells was defined as scMS ≥0.046 with a sensitivity of 88.2% and a specificity of 93.8%. The AUC of this model was 0.961, which was far beyond the AUC of each parameter alone (Figure 2B). In addition to current approaches, this method can surely aid in the analysis of single‐cell sequencing data in LUAD by better distinguishing malignant cells from other types.

### 3.3 Malignant differentiation trajectories uncover IRX2, SPINK13, and CAPN8 are important in tumor progression in LUAD

**FIGURE 3:**
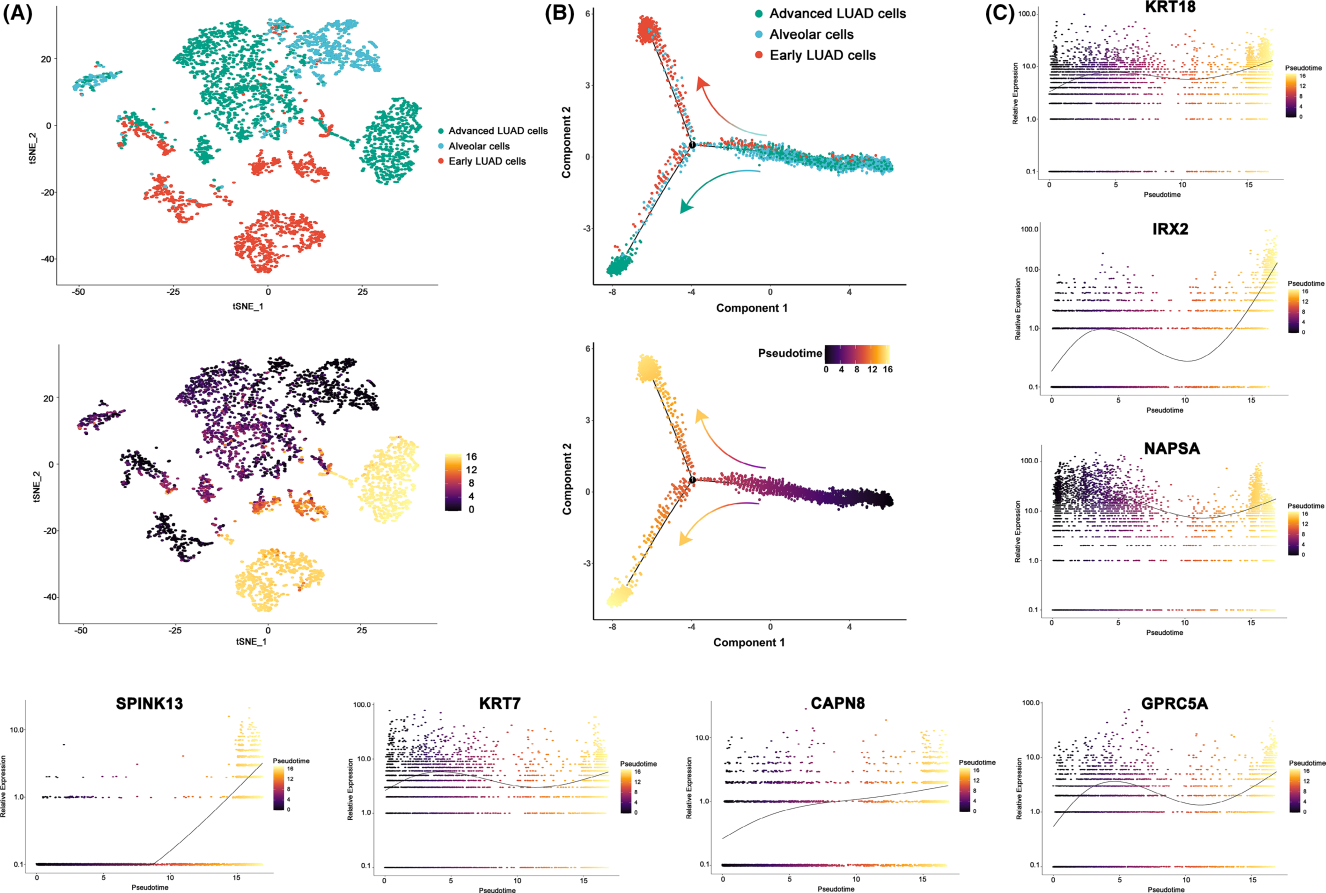
Transcriptional trajectory analysis regarding the seven final genes in tumor and alveolar cells from 17 tumor samples. (A) The transcriptional trajectory of cancer and alveolar cells. TOP: Cells with different colors indicating different cell type clusters identified in Seurat. Bottom: Cells colored by their assigned pseudotime values. (B) Reconstruction of pseudo‐temporal trajectories showing the progression of tumors. TOP: Cells with different colors showing different cell type clusters identified in Seurat. Bottom: Cells colored by their assigned pseudotime values. (C) Jitter plots visualizing expression data for the seven genes changing with pseudotime values

Given that KRT18, IRX2, NAPSA, SPINK13, KRT7, CAPN8, and GPRC5A could preferentially express in single malignant cells, we, therefore, went into the roles that they might play in LUAD. First, we extracted cells from all 17 tumor tissues (11 early‐stage and six advanced‐stage tumors) and constructed a transcriptional trajectory (Figure 3A). We aimed to figure out whether these seven genes had similar effects on tumor biology or not in different stages of LUAD.

Cells were first ordered according to their progression state using the monocle3 R package. Then, we manually set the start of pseudotime within alveolar cells before calculating the pseudotime value of each cancer cell by differential expression analysis. Cancer cells were located in two separate trajectory branches, marking their distinct differentiation state, as demonstrated in Figure 3B.

Transcriptomic and clinical colocalization atlas showed distinct gene expression profiles among different tumor stages. KRT18, IRX2, NAPSA, SPINK13, KRT7, CAPN8, and GPRC5A were all upregulated in tumors or advanced tumors (Figure 3C). IRX2 and SPINK13 were the most remarkable. Each showed a sharp increase in advanced cancer cells compared to normal alveolar or early cancer cells, indicating that they were all possibly key genes governing the tumor progression in LUAD.

Some of these genes have been well characterized in lung cancer, for instance, NAPSA^29^ is a common prognostic marker for LUAD, and KRT18, KRT7, and GPRC5A have been reported to play important roles in tumor progression and chemoresistance in various types of cancers.^30^, ^31^, ^32^, ^33^, ^34^, ^35^, ^36^, ^37^ Finally, we decided to focus on the functions of IRX2, SPINK13, and CAPN8, which have been implicated and not fully explored.

### 3.4 IRX2, SPINK13, and CAPN8 express high at both tissue and cell levels and promote proliferation in LUAD cells

**FIGURE 4:**
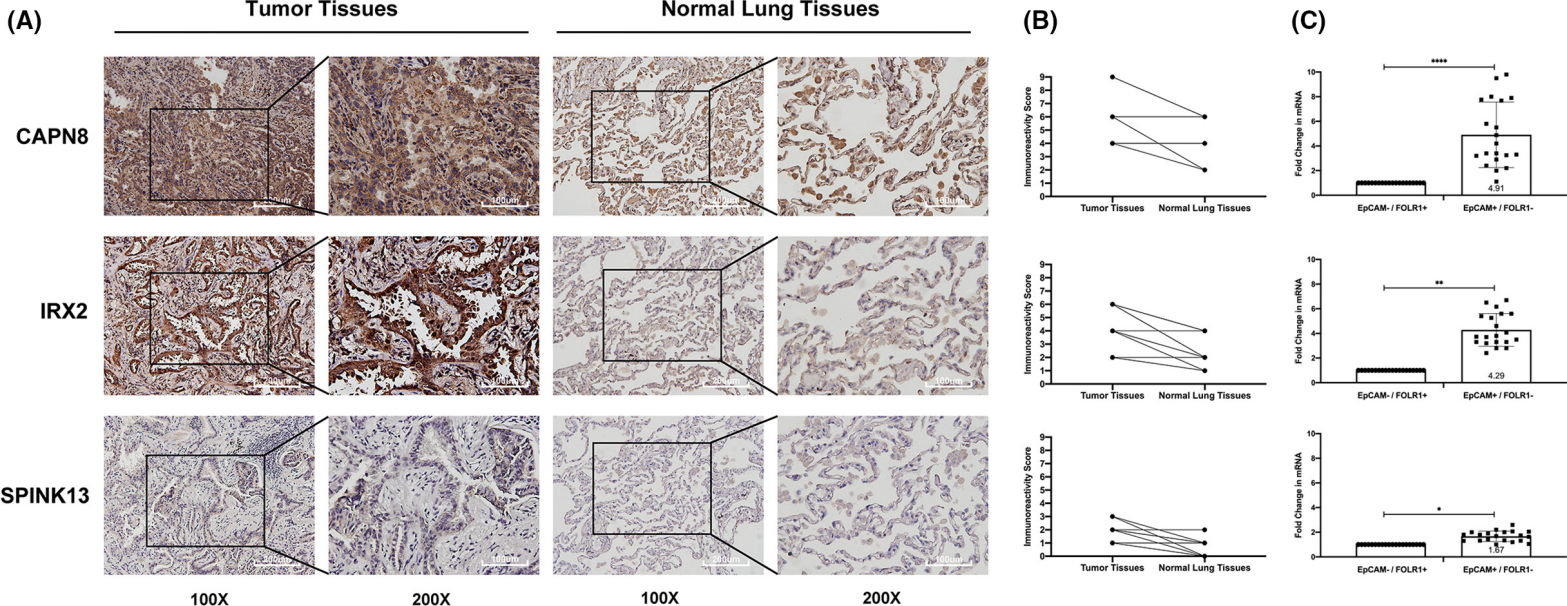
Expression levels of CAPN8, IRX2, and SPINK13 at tissue and cellular levels. (A) Representative images of the 20 LUAD patients showed the elevation of CAPN8, IRX2, SPINK13 expression in tumor samples compared to normal lungs in the same patient analyzed with IHC. (B) IRS scores illustrating the staining intensity of colored cells in each of the 20 IHC images in pairs. *N* = 20 patients (black dots). (C) Expression of levels of CAPN8, IRX2, and SPINK13 resulting from qRT‐PCR in two groups of sorted cells (EpCAM+/FOLR1– and EpCAM–/FOLR1+ cells) by flow cytometry. Data are shown as the mean ± SD from 20 experiments using samples collected from 20 LUAD patients. *p < 0.05; **p < 0.01; ****p < 0.0001 (Student's *t*‐test)

As first‐proof‐of‐principle functional validation experiments, we validated the expression status of IRX2, SPINK13, and CAPN8 at both tissue and cellular levels with specimen tissues from another 20 patients with primary LUAD before exploring their roles. Results from IHC achieved high concordance with the scRNA‐seq data. These three genes were confirmed to be expressed higher in tumors when compared to normal lungs, where CAPN8 expressed relatively the highest among the three genes (Figure 4A).

Their expression status in different types of cells was also measured using qRT‐PCR after conducting an EPCAM/FOLR1‐based flow cytometric analysis and cell sorting with another 20 pairs of specimen tissues from patients with LUAD as we previously reported.^18^ Results in the qRT‐PCR using the two groups of sorted cells revealed that the expression of either IRX2 (*p* < 0.01), SPINK13 (*p* < 0.05), or CAPN8 (*p* < 0.0001) was significantly higher in EpCAM+/FOLR1– cells (designated as malignant cells) than in EpCAM–/FOLR1+ cells (defined as non‐malignant cells) (Figure 4C).

**FIGURE 5:**
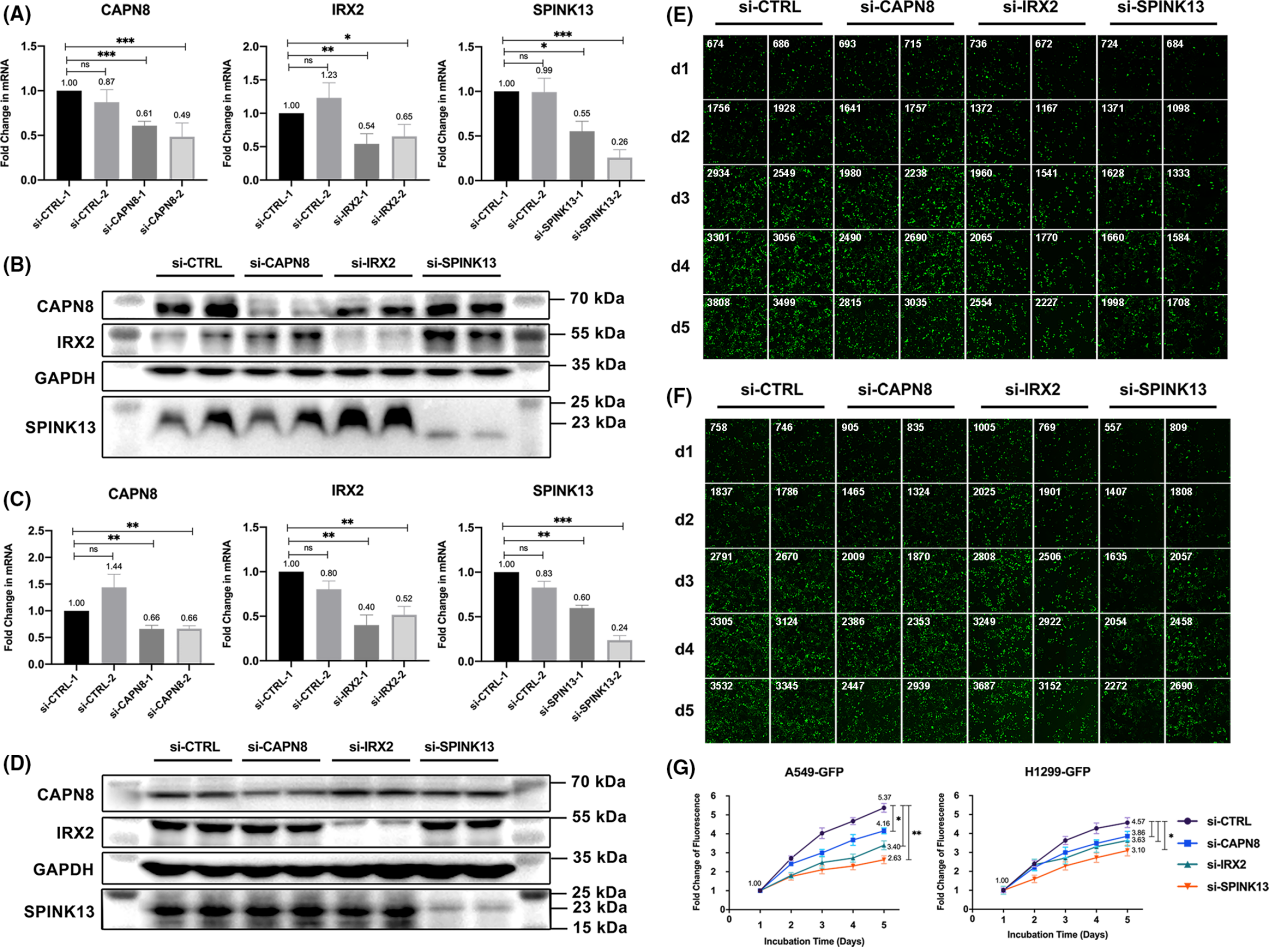
Effects of CAPN8, IRX2, and SPINK13 on cell proliferation in LUAD cell lines. (A) The three genes' expression levels by qRT‐PCR after silencing with different siRNAs in A549 transfected with GFP (A549‐GFP) cells for 24 h. Data are shown as the mean ± SD from three technical replicates (*n* = 3). (B) Western blot for the three genes in A549‐GFP cells transfected with different siRNAs for 48 h. (C) The three genes' expression levels by qRT‐PCR after silencing with different siRNAs in H1299 transfected with GFP (H1299‐GFP) cells for 24 h. Data are shown as the mean ± SD from three technical replicates (*n* = 3). (D) Western blot for the three genes in NCI‐H1299‐GFP cells transfected with different siRNAs for 48 h. (E) Fluorescence intensity and numbers of cells in each day after gene silencing in A549‐GFP cells. Data are shown as the mean ± SD from two biological replicates (*n* = 2). (F) Fluorescence intensity and numbers of cells in each day after gene silencing in NCI‐H1299‐GFP cells. Data are shown as the mean ± SD from two biological replicates (*n* = 2). (G) Fold changes of fluorescence intensity after gene silencing in A549‐GFP and NCI‐H1299‐GFP cells. Data are shown as the mean ± SD from two biological replicates (*n* = 2). *p < 0.05; **p < 0.01; ***p < 0.001; ns: Nonsignificance (Student's *t*‐test)

We then decided to simply investigate the functions of IRX2, SPINK13, and CAPN8 using two LUAD cell lines (A549 and NCI‐H1299). In vitro, after successfully transfected with GFP, we silenced IRX2, SPINK13, and CAPN8 at both mRNA and protein levels in both cell lines with two siRNAs each to avoid off‐target effects. Results of qRT‐PCR and western blotting are displayed in Figure 5A–D.

Speculating that they were important modulators in tumor growth, cell proliferation assays were conducted by monitoring fluorescence intensity each day after the siRNA transfection in those knocked‐down cells. We found that transient, siRNA‐mediated suppression of either IRX2 (*p* < 0.01), SPINK13 (*p* < 0.01), or CAPN8 (*p* < 0.05) led to a significantly attenuated proliferation capacity in A549 and a slight decrease (all three genes *p* < 0.05) in NCI‐H1299. Notably, SPINK13 had the strongest effect on cell proliferation, followed by IRX2 and CAPN8 in both cell lines (Figure 5E,G).

**FIGURE 6:**
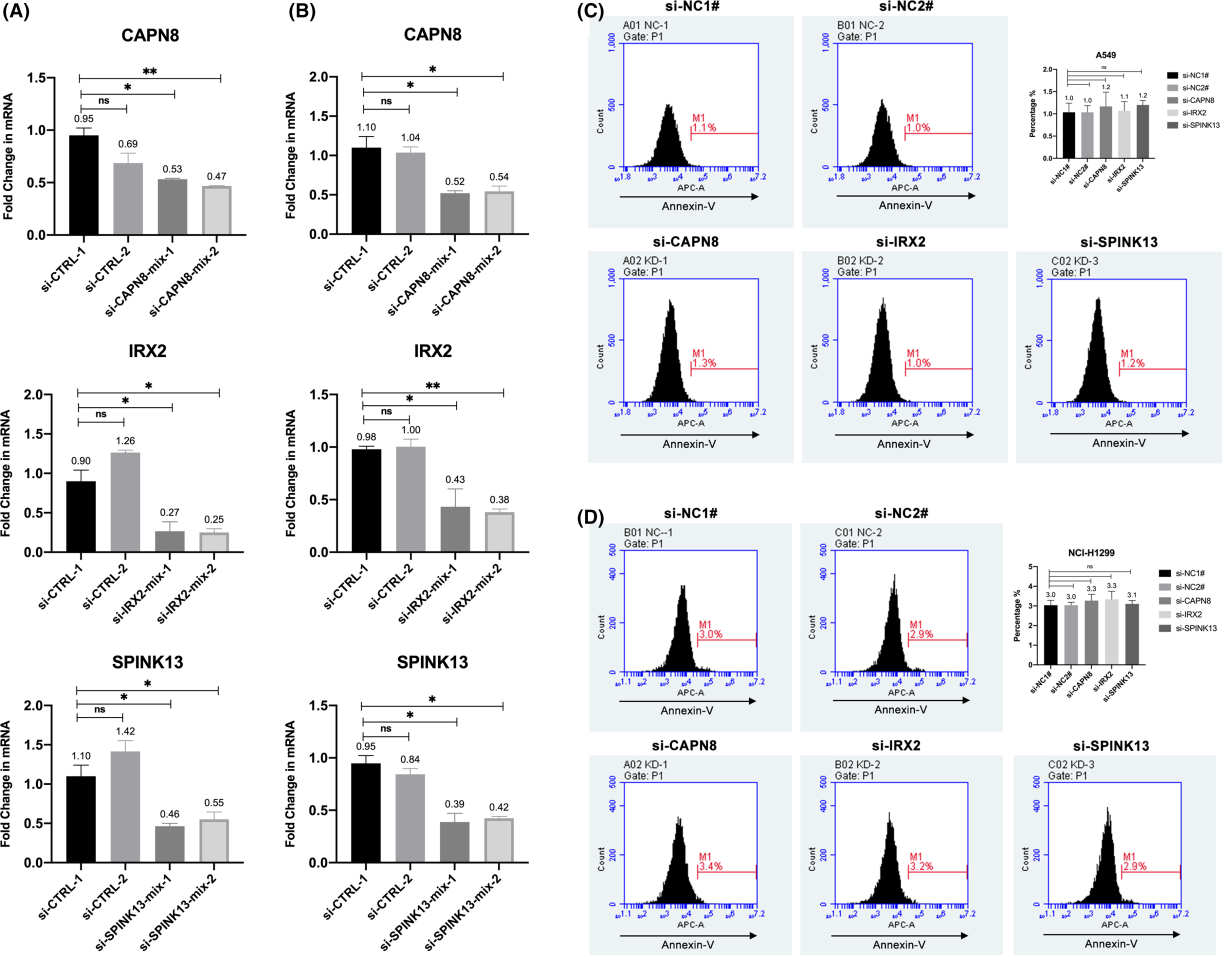
Effects of CAPN8, IRX2, and SPINK13 on cell death in A549 and NCI‐H1299 cell lines. (A) The three genes' expression levels by qRT‐PCR after silencing with mixed siRNAs in A549 transfected with GFP (A549‐GFP) cells for 48 h. Data are shown as the mean ± SD from two biological replicates (*n* = 2). (B) The three genes' expression levels by qRT‐PCR after silencing with mixed siRNAs in NCI‐H1299 transfected with GFP (H1299‐GFP) cells for 48 h. Data are shown as the mean ± SD from two biological replicates (*n* = 2). (C) Percentages of annexin V^+^ populations after gene silencing in A549 cell lines. Left: Representative original histogram overlays of fluorescence reflecting apoptosis. Top right: Quantification of annexin V^+^ populations are shown as the mean ± SD from three biological replicates (*n* = 3). (D) Percentages of annexin V^+^ populations after gene silencing in NCI‐H1299 cell lines. Left: Representative original histogram overlays of fluorescence reflecting apoptosis. Top right: Quantification of annexin V^+^ populations are shown as the mean ± SD from three biological replicates (*n* = 3). *p < 0.05; **p < 0.01; ns: Nonsignificance (Student's *t*‐test)

In addition, to better elucidate whether targeting CAPN8, IRX2, and SPINK13 could lead to cell death other than slowing down the proliferation, we conducted an Annexin V staining. After successfully knocking down each of the three genes by approximately 50% using mixed siRNAs at a final concentration of 100 nM, Annexin V^+^ cell populations were similar to the number in the control groups (Figure 6). Our results suggested that targeting CAPN8, IRX2, or SPINK13 hardly exerted a cytotoxic effect on A549 and NCI‐H1299 cells.

Collectively, the expression status of IRX2, SPINK13, and CAPN8 was in line with our findings in scRNA‐seq that they all showed an elevation both in tumor tissues and cells, which was also coincided with the development of tumor stage in LUAD. In addition, they could promote the proliferation of A549 and NCI‐H1299 cells, raising the possibility that they contributed to the tumor progression. However, targeting CAPN8, IRX2, or SPINK13 hardly exerted a cytotoxic effect on LUAD in vitro. Our results signaled that IRX2, SPINK13, and CAPN8 could serve as novel therapeutic targets to prevent tumor growth in LUAD.

## 4 DISCUSSION

This study investigated the gene expression profile of single cells based on the scRNA‐seq data of more than 200,000 cells from 29 samples in LUAD. After successfully identifying 369 preferentially expressed genes in single malignant cells, we explored their functions using gene enrichment analyses. They were highly associated with recurrence and oncogenesis. They also participated in the tumor microenvironment associated with cell adhesion and communications. Our results revealed that 293 of the 369 genes were upregulated in single malignant cells. As a result, their preferential elevation could make them attractive therapeutic targets in LUAD, rendering targeted drugs safer and more effective by protecting the other types of cells from being killed.

Besides, seven markedly correlated predictors (KRT18, IRX2, NAPSA, SPINK13, KRT7, CAPN8, and GPRC5A) for single malignant cells were selected from the 369 genes using the LASSO regression method in the present study. We found that they outperformed most of the other common predictors in distinguishing tumor cells in epithelial cancers. Therefore, we integrated them to generate a more accurate statistic model to predict a cell type within scRNA‐seq data. We finally introduced a score, scMS, calculated by the abundance of the seven genes, which could predict the malignancy of every single cell.

Previously developed approaches, such as “inferCNV”, have made it possible to estimate genomic copy number profiles from RNA read counts. Its principle is to identify aneuploid copy number profiles that are highly common in most human tumors.^10^, ^38^ However, “inferCNV” cannot accurately resolve chromosome breakpoints based on an average moving window of gene expression. As an alternative, the present model linked and integrated the most prevalent methods and canonical markers used in single‐cell clustering with good accuracy. We expected that it would be a valuable tool to quickly pick out single tumor cells from certain single‐cell data so they can be studied independently. In addition, we considered that the current model could also be suitable for pathological screenings as it combined seven predictors, all of which were relatively highly expressed in tumor cells.

Moreover, we found that IRX2, SPINK13, and CAPN8 showed great superiority to their counterparts in differentiation, especially in the ability to distinguish malignant tumor cells from normal epithelial cells. Transcriptional trajectory analysis consistently revealed their relevance to tumor progression in LUAD. We also detected their expression in lung adenocarcinoma samples using immunohistochemical staining and flow cytometry. Results confirmed their preferentially high expression in malignant cells. Subsequent experiments in vitro also uncovered their potential roles in promoting tumor proliferation in LUAD.

One of the highlights of our study was that we identified several tumor‐related genes that were scarcely revealed through analyses of bulk data. This was partly attributed to the application of scRNA‐seq technology. Because tumor samples used for transcriptome sequencing are mixtures of different cell populations, traditional microarray and RNA sequencing can simply detect the average gene abundance from a sample where cancer cells often account for a small proportion.^18^ In contrast, single‐cell sequencing technologies, such as 10X Genomics, can effectively explore the expression profile in each cell type, making it possible to dissect cellular heterogeneity and access unobtainable biological information from bulk analyses. Based on analyses of cells at a single‐cell level, we identified a few genes that had been rarely noticed before. They may have potential research and clinical utility in LUAD, which added understanding of complex cell biological processes and addressed heterogeneity across different cell populations.

The present study uncovered that high expression of IRX2, SPINK13, and CAPN8 could facilitate tumor progression in LUAD with multiple lung adenocarcinoma cell lines. Compared to previous studies associated with tumor biology, there are only a few controversies on these three genes. Regarding IRX2, Karlsson et al.^39^ reported that IRX2 was highly expressed in kidney clear cell sarcoma. Liu et al.^40^, ^41^ revealed that IRX2 could promote cell proliferation and invasion via Akt‐mediated upregulation of MMP9 and VEGF in osteosarcoma. Werner et al.^42^ reported that IRX2 inhibited cellular motility and chemokine expression in breast cancer cells.

Concerning SPINK13, Xu et al.^43^ found that higher SPINK13 expression was associated with worse survival in patients with renal cell carcinoma. According to their bioinformatics analyses, it might be involved in epithelial‐mesenchymal transition (EMT), glycolysis, hypoxia, and inflammation signaling pathways. Accordantly, Cai et al.^44^ and Wei et al.^45^ reported that SPINK13 could inhibit tumor growth and metastasis via urokinase‐type plasminogen activator in ovarian cancer and hepatocellular carcinoma. As for CAPN8, Chen et al.^46^ found that downregulation of CAPN8 was associated with the increased smoking‐related mutations in lung adenocarcinoma. Ma et al.^47^ constructed a seven‐gene signature to predict lung squamous cell carcinoma prognosis and identified CAPN8 as a hub gene in the signature.

Taking the above results and ours together, we can conclude that we have preliminarily determined the role of IRX2, SPINK13, and CAPN8 in LUAD, making them more likely to be used as therapeutic targets for tumor therapy. However, there are still two issues that should be pointed out if the three genes are used in clinical practice. According to current evidence, silencing them could only inhibit cell growth but cannot kill cancer cells. Therefore, CAPN8, IRX2, and SPINK13 should be targeted with other combined treatments. On the other hand, SPINK13 was the best, whether in terms of discerning tumor cells, its correlation with advanced tumors, or promoting proliferation in vitro. However, neither of its expression in single tumor cells or LUAD tissues was satisfied, which we considered was attributed to its feature that SPINK13 was a secreted protein. If it were to be applied in clinical practice, therapeutic agents targeting this gene with strong specificity should be developed.

Furthermore, among the 369 specifically expressed genes in single tumor cells, we took a deep look into the 70 genes encoding secreted or membrane‐bound proteins. Since proteins have sparked fast‐growing interest as biological therapeutic agents for several diseases, these genes are promising targets in anticancer therapies. By building CAR T cells with their cancer‐specific antigens or simply coupling their antibodies with chemotherapeutic reagents, they can be used to guide drugs to kill tumor cells more specifically than the current.

For instance, our analyses revealed that MUC1 could be used for distinguishing single tumor cells and normal cells with the highest AUC (0.916) among all the 70 genes. At the same time, its potential in immunotherapy had just been frequently reported. One of the previous studies demonstrated the target‐specific cytotoxicity of anti‐Tn‐MUC1 (Tn glycol‐form of MUC1) CAR T cells, which could successfully control tumor growth.^48^ Besides, MUC1 has also been involved in therapeutic cancer vaccines in recent years. In lung cancer, TG4010, a modified vaccine targeting MUC1 and interleukin 2, showed improvements in progression‐free survival compared to placebo in a phase 2b/3 trial.^49^ An adenovirus vaccine targeting prostate‐specific antigen, brachyury, and MUC1 together was also used in Phase I clinical trials in prostate cancer, ending with good tolerance.^50^ Other membrane proteins in our study have set forth their roles in immunology and potentials in immunotherapies, where CD24 has recently attracted the growing interest of researchers.^51^

This study investigated single‐cell sequencing data from lung adenocarcinoma samples and successfully identified genes preferentially expressed in single malignant cells. The roles of all these genes, including those encoding secreted or membrane‐bound proteins, had been associated with tumor biology. We also constructed a seven‐gene‐based prediction model to accurately distinguish single malignant cells from other types in lung adenocarcinoma. Three genes in the model, IRX2, SPINK13, and CAPN8, were also validated with their expression and potentials in tumor proliferation. Our results signaled that IRX2, SPINK13, and CAPN8 could serve as novel therapeutic targets to prevent tumor growth in lung adenocarcinoma.

There are still some limitations in our study. A notable limitation is that since only 29 samples from patients with LUAD were used for sequencing, the genes that we identified that can discern tumor cells need to be validated with more scRNA data using extra samples. Besides, the genes included in the present prediction model cannot be expressed in all types of tumors at an appropriate level. Therefore, scRNA‐seq data of more types of tumors are needed to build similar suitable tools. Besides, we have to point out that only approximately 2000 genes in total were detected in our data for analyses because of the relatively low expression of some genes in single cells. Apparently, this limitation can lead to an increasing underestimation of certain genes. Last but not least, genes identified as potential therapeutic targets in the present study still need to be validated in more in vitro and in vivo. We hope our findings can expedite the transformation from data analytics to personalized clinical medicine.

## CONFLICT OF INTEREST

The authors have no conflict of interest to declare.

## ETHICS STATEMENT

This study was approved by the Ethics Committee of Zhongshan Hospital, Fudan University, China (B2018–137R). Informed consent was obtained when patients were hospitalized.

## AUTHOR CONTRIBUTIONS

J.L., C.Z. and Q.W. conceived and designed the research. J.L., Y.H. and G.B. collected specimen samples. J.L., G.B. and Z.C. analyzed data and provided interpretation. Q.S., and X.J. provided technical support. J.L., Y.B., X.J. and H.Z. participated in the evaluation of results. J.L., C.Z. and Q.W. wrote, reviewed and revised the manuscript, and all authors reviewed and approved the manuscript for publication..

## Supporting information


Table S1
Table S2
Table S3
Table S4

## Data Availability

The datasets used and/or analyzed during the current study are available from the corresponding author on reasonable request.
