# Supplementary material for: Signatures of malignant cells and novel therapeutic targets revealed by single‐cell sequencing in lung adenocarcinoma
Source: Cancer Med. 2022 Jan 31;11(11):2244–58. doi: 10.1002/cam4.4547 (PMC9160812; doi:10.1002/cam4.4547)
Supplement: Supplementary file 1 — Table S1 Table S2 Table S3 Table S4 [file CAM4-11-2244-s001.docx]

# Table 1. Baseline characteristics of patients included in IHC

| No. | Age  (Years) | Sex | Smoking  Status | Histology | Stage | Activating  EGFR mutation | Previous  therapies |
| --- | --- | --- | --- | --- | --- | --- | --- |
| n = 20 | 60.5±7.5 | Male (52.4%)  Female (47.8%) | Current (10%)  Former (10%)  Never (80%) | LUAD (100%) | Early (75%)  Advanced (25%) | Negative (60%)  Positive (40%) | None (95%)  Immune-based (5%) |
| Patient 1# | 61 | Female | Never | LUAD | II | Positive | None |
| Patient 2# | 52 | Female | Never | LUAD | II | Negative | None |
| Patient 3# | 67 | Male | Former | LUAD | III (Advanced) | Negative | None |
| Patient 4# | 53 | Female | Never | LUAD | II | Positive | None |
| Patient 5# | 57 | Female | Never | LUAD | I | Negative | None |
| Patient 6# | 58 | Male | Never | LUAD | I | Negative | None |
| Patient 7# | 71 | Male | Current | LUAD | III(Advanced) | Negative | None |
| Patient 8# | 73 | Male | Never | LUAD | II | Negative | None |
| Patient 9# | 67 | Male | Never | LUAD | III(Advanced) | Negative | None |
| Patient 10# | 73 | Male | Former | LUAD | I | Positive | None |
| Patient 11# | 64 | Female | Never | LUAD | I | Negative | None |
| Patient 12# | 56 | Male | Never | LUAD | I | Negative | None |
| Patient 13# | 59 | Female | Never | LUAD | I | Negative | None |
| Patient 14# | 67 | Female | Never | LUAD | II | Positive | None |
| Patient 15# | 53 | Male | Current | LUAD | III(Advanced) | Positive | Immune-based |
| Patient 16# | 45 | Female | Never | LUAD | I | Negative | None |
| Patient 17# | 57 | Male | Never | LUAD | II | Positive | None |
| Patient 18# | 61 | Male | Never | LUAD | III(Advanced) | Positive | None |
| Patient 19# | 58 | Female | Never | LUAD | II | Negative | None |
| Patient 20# | 66 | Male | Never | LUAD | III(Advanced) | Positive | None |

LUAD: Lung adenocarcinoma

# Table 2. siRNA targeting sequences

| No. | Source | Sequence |
| --- | --- | --- |
| si-CAPN8 1# | Homo sapiens | GAGAACTATGCGGGAATCTTTCA |
| si-CAPN8 2# | Homo sapiens | GCCAAGCTTAATGGTTGTTATGA |
| si-IRX2 1# | Homo sapiens | GCCTCAAGAAGGAGAACAAGATG |
| si-IRX2 2# | Homo sapiens | CACAAACTACGGGAACTTGAACG |
| si-SPINK13 1# | Homo sapiens | TGGCTTTCTCAGGAATTTTCAAT |
| si-SPINK13 2# | Homo sapiens | GCCACACTTTCCAGAATGAGTGT |

# Table 3. Primer Sequences of CAPN8, IRX2, SPINK13, and GAPDH gene

| Gene | Source | Sequence 5’ -> 3’ |
| --- | --- | --- |
| CAPN8 | Homo sapiens | Forward: TGGCTCCAACCAAAACGCTT |
|  |  | Reverse: CCTGGTCCAAGATCCTTGTAGC |
| IRX2 | Homo sapiens | Forward: CCTACCCGTACCAGCTCAAC |
|  |  | Reverse: CTTCGCTTTTGTTTCTCGGGG |
| SPINK13 | Homo sapiens | Forward: GGCCTAAGCCCCGATGTAAAA |
|  |  | Reverse: GGCCATTTGAGGCACAAACA |
| GAPDH | Homo sapiens | Forward: AGAAGGCTGGGGCTCATTTG |
|  |  | Reverse: AGGGGCCATCCACAGTCTTC |

# Table 4. Features of the 369 preferentially-expressed genes in single malignant cells

| Rank | Gene Symbol | AUC | Sensitivity | Specificity | \|log2FC\| | Relative expression in single malignant cells | Main subcellular locations of the protein |
| --- | --- | --- | --- | --- | --- | --- | --- |
| 1 | KRT18 | 0.927 | 0.903 | 0.865 | 1.855 | High | Cytosol, nucleoplasm, etc. |
| 2 | KRT8 | 0.924 | 0.839 | 0.923 | 1.851 | High | Cytosol, nucleoplasm, etc. |
| 3 | NAPSA | 0.921 | 0.899 | 0.881 | 1.994 | High | Cytosol, nucleoplasm, etc. |
| 4 | MUC1 | 0.916 | 0.907 | 0.861 | 1.729 | High | Secreted or membrane-bound |
| 5 | WFDC2 | 0.912 | 0.897 | 0.850 | 1.898 | High | Cytosol, nucleoplasm, etc. |
| 6 | SFTPB | 0.901 | 0.888 | 0.854 | 1.989 | High | Cytosol, nucleoplasm, etc. |
| 7 | HOPX | 0.900 | 0.879 | 0.827 | 1.693 | High | Cytosol, nucleoplasm, etc. |
| 8 | KRT7 | 0.892 | 0.867 | 0.865 | 1.468 | High | Cytosol, nucleoplasm, etc. |
| 9 | EPCAM | 0.885 | 0.885 | 0.837 | 1.485 | High | Secreted or membrane-bound |
| 10 | KRT19 | 0.884 | 0.839 | 0.867 | 1.413 | High | Cytosol, nucleoplasm, etc. |
| 11 | SFTA2 | 0.878 | 0.840 | 0.878 | 1.218 | High | Cytosol, nucleoplasm, etc. |
| 12 | ZFP36L1 | 0.877 | 0.812 | 0.828 | 1.232 | High | Cytosol, nucleoplasm, etc. |
| 13 | ERRFI1 | 0.874 | 0.842 | 0.831 | 1.671 | High | Cytosol, nucleoplasm, etc. |
| 14 | C8orf4 | 0.873 | 0.840 | 0.833 | 2.120 | High | Cytosol, nucleoplasm, etc. |
| 15 | ELF3 | 0.872 | 0.878 | 0.827 | 1.561 | High | Cytosol, nucleoplasm, etc. |
| 16 | NKX2-1 | 0.865 | 0.924 | 0.790 | 1.109 | High | Secreted or membrane-bound |
| 17 | RNASE1 | 0.865 | 0.830 | 0.824 | 1.169 | High | Secreted or membrane-bound |
| 18 | GPRC5A | 0.864 | 0.813 | 0.781 | 1.025 | High | Secreted or membrane-bound |
| 19 | CLDN4 | 0.862 | 0.879 | 0.807 | 1.247 | High | Secreted or membrane-bound |
| 20 | SLC34A2 | 0.861 | 0.875 | 0.822 | 1.461 | High | Secreted or membrane-bound |
| 21 | MGST1 | 0.861 | 0.769 | 0.887 | 1.116 | High | Cytosol, nucleoplasm, etc. |
| 22 | ATP1A1 | 0.855 | 0.835 | 0.764 | 1.116 | High | Cytosol, nucleoplasm, etc. |
| 23 | TACSTD2 | 0.855 | 0.887 | 0.793 | 1.091 | High | Secreted or membrane-bound |
| 24 | MT-ND1 | 0.853 | 0.904 | 0.777 | 1.317 | High | Cytosol, nucleoplasm, etc. |
| 25 | MAL2 | 0.847 | 0.915 | 0.760 | 1.046 | High | Cytosol, nucleoplasm, etc. |
| 26 | EMP2 | 0.847 | 0.826 | 0.815 | 0.846 | High | Cytosol, nucleoplasm, etc. |
| 27 | CYB5A | 0.842 | 0.814 | 0.777 | 1.149 | High | Cytosol, nucleoplasm, etc. |
| 28 | SFTA3 | 0.841 | 0.907 | 0.767 | 0.951 | High | Cytosol, nucleoplasm, etc. |
| 29 | WSB1 | 0.839 | 0.865 | 0.699 | 0.922 | High | Cytosol, nucleoplasm, etc. |
| 30 | LPCAT1 | 0.839 | 0.830 | 0.785 | 1.226 | High | Cytosol, nucleoplasm, etc. |
| 31 | ATP1B1 | 0.839 | 0.766 | 0.818 | 1.136 | High | Cytosol, nucleoplasm, etc. |
| 32 | SRGN | 0.836 | 0.700 | 0.921 | -2.554 | Low | Cytosol, nucleoplasm, etc. |
| 33 | NEAT1 | 0.836 | 0.783 | 0.764 | 1.497 | High | Cytosol, nucleoplasm, etc. |
| 34 | IRX2 | 0.835 | 0.950 | 0.701 | 1.734 | High | Cytosol, nucleoplasm, etc. |
| 35 | SPINT2 | 0.835 | 0.846 | 0.723 | 1.064 | High | Secreted or membrane-bound |
| 36 | ATP11A | 0.834 | 0.895 | 0.733 | 1.094 | High | Secreted or membrane-bound |
| 37 | MT-ATP6 | 0.833 | 0.775 | 0.759 | 0.861 | High | Cytosol, nucleoplasm, etc. |
| 38 | CLDN7 | 0.828 | 0.867 | 0.760 | 0.879 | High | Secreted or membrane-bound |
| 39 | HSPB1 | 0.827 | 0.779 | 0.759 | 0.730 | High | Secreted or membrane-bound |
| 40 | CD55 | 0.825 | 0.822 | 0.732 | 0.917 | High | Cytosol, nucleoplasm, etc. |
| 41 | LGALS3BP | 0.825 | 0.712 | 0.844 | 1.225 | High | Cytosol, nucleoplasm, etc. |
| 42 | S100A6 | 0.825 | 0.711 | 0.810 | 0.859 | High | Secreted or membrane-bound |
| 43 | MALL | 0.824 | 0.912 | 0.719 | 1.194 | High | Cytosol, nucleoplasm, etc. |
| 44 | SDC4 | 0.823 | 0.805 | 0.785 | 0.943 | High | Cytosol, nucleoplasm, etc. |
| 45 | TM4SF1 | 0.823 | 0.846 | 0.769 | 0.795 | High | Cytosol, nucleoplasm, etc. |
| 46 | CXCL17 | 0.822 | 0.895 | 0.740 | 1.010 | High | Cytosol, nucleoplasm, etc. |
| 47 | LMO7 | 0.822 | 0.900 | 0.723 | 0.920 | High | Cytosol, nucleoplasm, etc. |
| 48 | MT-CO3 | 0.821 | 0.754 | 0.763 | 0.615 | High | Cytosol, nucleoplasm, etc. |
| 49 | MYO6 | 0.821 | 0.898 | 0.717 | 0.870 | High | Cytosol, nucleoplasm, etc. |
| 50 | RHOB | 0.819 | 0.772 | 0.762 | 1.050 | High | Cytosol, nucleoplasm, etc. |
| 51 | SDC1 | 0.818 | 0.908 | 0.705 | 1.223 | High | Cytosol, nucleoplasm, etc. |
| 52 | SMIM22 | 0.818 | 0.894 | 0.722 | 1.012 | High | Cytosol, nucleoplasm, etc. |
| 53 | SEPW1 | 0.817 | 0.753 | 0.762 | 0.854 | High | Cytosol, nucleoplasm, etc. |
| 54 | MBIP | 0.816 | 0.875 | 0.723 | 0.863 | High | Cytosol, nucleoplasm, etc. |
| 55 | SSR4 | 0.816 | 0.844 | 0.702 | 1.116 | High | Cytosol, nucleoplasm, etc. |
| 56 | BCAM | 0.814 | 0.910 | 0.700 | 0.943 | High | Cytosol, nucleoplasm, etc. |
| 57 | S100A13 | 0.812 | 0.738 | 0.817 | 0.997 | High | Secreted or membrane-bound |
| 58 | VEGFA | 0.811 | 0.836 | 0.735 | 1.257 | High | Cytosol, nucleoplasm, etc. |
| 59 | AGR2 | 0.811 | 0.889 | 0.718 | 1.081 | High | Cytosol, nucleoplasm, etc. |
| 60 | PIGR | 0.810 | 0.887 | 0.710 | 1.386 | High | Cytosol, nucleoplasm, etc. |
| 61 | IFI27 | 0.807 | 0.805 | 0.737 | 0.786 | High | Cytosol, nucleoplasm, etc. |
| 62 | TSPAN3 | 0.807 | 0.796 | 0.755 | 0.862 | High | Cytosol, nucleoplasm, etc. |
| 63 | FOLR1 | 0.806 | 0.895 | 0.694 | 1.259 | High | Cytosol, nucleoplasm, etc. |
| 64 | CAPN8 | 0.806 | 0.949 | 0.652 | 0.942 | High | Secreted or membrane-bound |
| 65 | APLP2 | 0.805 | 0.760 | 0.736 | 0.898 | High | Secreted or membrane-bound |
| 66 | SPTSSA | 0.804 | 0.782 | 0.764 | 0.950 | High | Cytosol, nucleoplasm, etc. |
| 67 | FXYD3 | 0.803 | 0.877 | 0.706 | 0.949 | High | Cytosol, nucleoplasm, etc. |
| 68 | EFNA1 | 0.801 | 0.903 | 0.689 | 0.748 | High | Cytosol, nucleoplasm, etc. |
| 69 | OCIAD2 | 0.800 | 0.755 | 0.753 | 0.687 | High | Cytosol, nucleoplasm, etc. |
| 70 | PHLDA2 | 0.800 | 0.811 | 0.730 | 1.090 | High | Cytosol, nucleoplasm, etc. |
| 71 | CEACAM6 | 0.800 | 0.923 | 0.664 | 1.239 | High | Cytosol, nucleoplasm, etc. |
| 72 | CEBPD | 0.799 | 0.760 | 0.743 | 1.060 | High | Cytosol, nucleoplasm, etc. |
| 73 | RASD1 | 0.798 | 0.905 | 0.661 | 1.701 | High | Cytosol, nucleoplasm, etc. |
| 74 | GNAS | 0.797 | 0.814 | 0.669 | 1.260 | High | Cytosol, nucleoplasm, etc. |
| 75 | VMP1 | 0.797 | 0.886 | 0.617 | 1.057 | High | Cytosol, nucleoplasm, etc. |
| 76 | CCND1 | 0.797 | 0.890 | 0.679 | 1.048 | High | Cytosol, nucleoplasm, etc. |
| 77 | HMGB3 | 0.796 | 0.904 | 0.659 | 1.353 | High | Cytosol, nucleoplasm, etc. |
| 78 | DPP4 | 0.796 | 0.950 | 0.629 | 0.926 | High | Cytosol, nucleoplasm, etc. |
| 79 | SLC22A31 | 0.796 | 0.943 | 0.645 | 0.862 | High | Cytosol, nucleoplasm, etc. |
| 80 | S100A14 | 0.795 | 0.899 | 0.690 | 0.784 | High | Secreted or membrane-bound |
| 81 | MTUS1 | 0.790 | 0.912 | 0.656 | 0.833 | High | Cytosol, nucleoplasm, etc. |
| 82 | LMO3 | 0.790 | 0.945 | 0.626 | 0.964 | High | Cytosol, nucleoplasm, etc. |
| 83 | CLPTM1L | 0.789 | 0.871 | 0.671 | 0.814 | High | Cytosol, nucleoplasm, etc. |
| 84 | SOX4 | 0.789 | 0.786 | 0.748 | 0.863 | High | Cytosol, nucleoplasm, etc. |
| 85 | PLS3 | 0.788 | 0.890 | 0.668 | 1.025 | High | Secreted or membrane-bound |
| 86 | SRRM2 | 0.788 | 0.818 | 0.685 | 0.532 | High | Cytosol, nucleoplasm, etc. |
| 87 | ASPH | 0.788 | 0.825 | 0.705 | 1.023 | High | Cytosol, nucleoplasm, etc. |
| 88 | AQP3 | 0.788 | 0.795 | 0.726 | 0.984 | High | Secreted or membrane-bound |
| 89 | JUND | 0.788 | 0.788 | 0.701 | 0.750 | High | Cytosol, nucleoplasm, etc. |
| 90 | GSTP1 | 0.786 | 0.762 | 0.693 | 0.695 | High | Cytosol, nucleoplasm, etc. |
| 91 | ZDHHC9 | 0.785 | 0.939 | 0.619 | 0.755 | High | Cytosol, nucleoplasm, etc. |
| 92 | TMC5 | 0.784 | 0.936 | 0.630 | 0.754 | High | Secreted or membrane-bound |
| 93 | MPZL2 | 0.782 | 0.911 | 0.641 | 0.748 | High | Cytosol, nucleoplasm, etc. |
| 94 | C16orf89 | 0.782 | 0.905 | 0.647 | 1.054 | High | Cytosol, nucleoplasm, etc. |
| 95 | PRSS8 | 0.782 | 0.948 | 0.610 | 0.746 | High | Cytosol, nucleoplasm, etc. |
| 96 | RAB11FIP1 | 0.781 | 0.789 | 0.700 | 1.155 | High | Secreted or membrane-bound |
| 97 | CDH1 | 0.781 | 0.945 | 0.610 | 0.764 | High | Secreted or membrane-bound |
| 98 | S100A10 | 0.781 | 0.658 | 0.796 | 0.779 | High | Cytosol, nucleoplasm, etc. |
| 99 | PPP1R14B | 0.780 | 0.820 | 0.706 | 0.740 | High | Cytosol, nucleoplasm, etc. |
| 100 | C4BPA | 0.778 | 0.934 | 0.613 | 1.260 | High | Cytosol, nucleoplasm, etc. |
| 101 | NEDD4L | 0.777 | 0.924 | 0.619 | 0.758 | High | Cytosol, nucleoplasm, etc. |
| 102 | MGLL | 0.777 | 0.880 | 0.649 | 0.799 | High | Cytosol, nucleoplasm, etc. |
| 103 | CD52 | 0.776 | 0.630 | 0.869 | -2.293 | Low | Cytosol, nucleoplasm, etc. |
| 104 | P4HB | 0.774 | 0.746 | 0.720 | 0.646 | High | Cytosol, nucleoplasm, etc. |
| 105 | DUSP6 | 0.774 | 0.823 | 0.669 | 0.913 | High | Cytosol, nucleoplasm, etc. |
| 106 | PRDX5 | 0.773 | 0.798 | 0.670 | 0.550 | High | Cytosol, nucleoplasm, etc. |
| 107 | LSR | 0.772 | 0.914 | 0.619 | 0.671 | High | Cytosol, nucleoplasm, etc. |
| 108 | FAM13A | 0.772 | 0.926 | 0.604 | 0.876 | High | Cytosol, nucleoplasm, etc. |
| 109 | NPC2 | 0.771 | 0.657 | 0.794 | 0.965 | High | Cytosol, nucleoplasm, etc. |
| 110 | ADGRF5 | 0.771 | 0.911 | 0.624 | 0.780 | High | Cytosol, nucleoplasm, etc. |
| 111 | C5orf38 | 0.771 | 0.950 | 0.583 | 0.784 | High | Secreted or membrane-bound |
| 112 | DSTN | 0.769 | 0.736 | 0.740 | 0.525 | High | Cytosol, nucleoplasm, etc. |
| 113 | TOP1 | 0.769 | 0.696 | 0.754 | 0.644 | High | Cytosol, nucleoplasm, etc. |
| 114 | TXNDC17 | 0.769 | 0.831 | 0.607 | 0.873 | High | Cytosol, nucleoplasm, etc. |
| 115 | RUNX1 | 0.768 | 0.786 | 0.705 | 0.661 | High | Cytosol, nucleoplasm, etc. |
| 116 | CP | 0.768 | 0.962 | 0.565 | 1.635 | High | Cytosol, nucleoplasm, etc. |
| 117 | MT-ND5 | 0.768 | 0.794 | 0.639 | 0.501 | High | Cytosol, nucleoplasm, etc. |
| 118 | SFN | 0.766 | 0.923 | 0.605 | 0.776 | High | Cytosol, nucleoplasm, etc. |
| 119 | CANX | 0.766 | 0.801 | 0.626 | 0.594 | High | Cytosol, nucleoplasm, etc. |
| 120 | CLDN3 | 0.765 | 0.930 | 0.591 | 0.919 | High | Cytosol, nucleoplasm, etc. |
| 121 | CD46 | 0.763 | 0.721 | 0.721 | 0.627 | High | Secreted or membrane-bound |
| 122 | PGRMC1 | 0.763 | 0.809 | 0.672 | 0.834 | High | Cytosol, nucleoplasm, etc. |
| 123 | EPS8 | 0.762 | 0.904 | 0.607 | 0.703 | High | Cytosol, nucleoplasm, etc. |
| 124 | LAPTM5 | 0.762 | 0.598 | 0.878 | -1.723 | Low | Cytosol, nucleoplasm, etc. |
| 125 | PON2 | 0.761 | 0.849 | 0.652 | 0.670 | High | Cytosol, nucleoplasm, etc. |
| 126 | PTPRF | 0.761 | 0.931 | 0.587 | 0.605 | High | Cytosol, nucleoplasm, etc. |
| 127 | ABCC3 | 0.761 | 0.908 | 0.601 | 0.785 | High | Secreted or membrane-bound |
| 128 | CTTN | 0.760 | 0.887 | 0.626 | 0.642 | High | Secreted or membrane-bound |
| 129 | CAPN2 | 0.760 | 0.723 | 0.721 | 0.502 | High | Cytosol, nucleoplasm, etc. |
| 130 | RAB25 | 0.759 | 0.929 | 0.583 | 0.680 | High | Cytosol, nucleoplasm, etc. |
| 131 | KLF5 | 0.758 | 0.936 | 0.573 | 0.671 | High | Cytosol, nucleoplasm, etc. |
| 132 | DMKN | 0.758 | 0.912 | 0.601 | 0.545 | High | Cytosol, nucleoplasm, etc. |
| 133 | C19orf33 | 0.757 | 0.904 | 0.604 | 0.866 | High | Cytosol, nucleoplasm, etc. |
| 134 | SFTPA2 | 0.757 | 0.799 | 0.674 | 0.868 | High | Cytosol, nucleoplasm, etc. |
| 135 | TFCP2L1 | 0.755 | 0.954 | 0.546 | 0.935 | High | Cytosol, nucleoplasm, etc. |
| 136 | CRNDE | 0.755 | 0.921 | 0.588 | 0.559 | High | Cytosol, nucleoplasm, etc. |
| 137 | LAPTM4B | 0.754 | 0.910 | 0.587 | 0.655 | High | Cytosol, nucleoplasm, etc. |
| 138 | CTNNA1 | 0.753 | 0.781 | 0.673 | 0.696 | High | Secreted or membrane-bound |
| 139 | KCNQ1OT1 | 0.753 | 0.810 | 0.653 | 0.737 | High | Cytosol, nucleoplasm, etc. |
| 140 | SERPINH1 | 0.753 | 0.819 | 0.676 | 0.525 | High | Cytosol, nucleoplasm, etc. |
| 141 | FAM129B | 0.753 | 0.900 | 0.589 | 0.678 | High | Secreted or membrane-bound |
| 142 | TANC2 | 0.753 | 0.914 | 0.577 | 0.712 | High | Cytosol, nucleoplasm, etc. |
| 143 | EGR1 | 0.753 | 0.836 | 0.591 | 1.011 | High | Cytosol, nucleoplasm, etc. |
| 144 | CTSH | 0.752 | 0.634 | 0.800 | 0.907 | High | Secreted or membrane-bound |
| 145 | PCSK1N | 0.752 | 0.960 | 0.538 | 0.791 | High | Secreted or membrane-bound |
| 146 | MARCH6 | 0.752 | 0.819 | 0.637 | 0.516 | High | Cytosol, nucleoplasm, etc. |
| 147 | SCGB3A1 | 0.751 | 0.677 | 0.733 | 1.939 | High | Cytosol, nucleoplasm, etc. |
| 148 | SLC20A1 | 0.751 | 0.818 | 0.623 | 1.348 | High | Secreted or membrane-bound |
| 149 | TIPARP | 0.751 | 0.809 | 0.642 | 0.953 | High | Cytosol, nucleoplasm, etc. |
| 150 | CXCR4 | 0.750 | 0.526 | 0.946 | -2.376 | Low | Cytosol, nucleoplasm, etc. |
| 151 | MLPH | 0.750 | 0.896 | 0.602 | 0.567 | High | Cytosol, nucleoplasm, etc. |
| 152 | WBP5 | 0.750 | 0.774 | 0.697 | 0.630 | High | Cytosol, nucleoplasm, etc. |
| 153 | SNHG25 | 0.749 | 0.804 | 0.646 | 0.787 | High | Cytosol, nucleoplasm, etc. |
| 154 | RBPMS | 0.749 | 0.911 | 0.575 | 0.678 | High | Cytosol, nucleoplasm, etc. |
| 155 | LLGL2 | 0.749 | 0.942 | 0.546 | 0.682 | High | Cytosol, nucleoplasm, etc. |
| 156 | ITGA3 | 0.749 | 0.942 | 0.550 | 0.602 | High | Secreted or membrane-bound |
| 157 | CTNND1 | 0.747 | 0.877 | 0.603 | 0.514 | High | Secreted or membrane-bound |
| 158 | ATF3 | 0.747 | 0.733 | 0.693 | 1.000 | High | Cytosol, nucleoplasm, etc. |
| 159 | AGR3 | 0.747 | 0.904 | 0.591 | 0.528 | High | Cytosol, nucleoplasm, etc. |
| 160 | LPP | 0.746 | 0.803 | 0.648 | 0.644 | High | Cytosol, nucleoplasm, etc. |
| 161 | PDCD6 | 0.745 | 0.887 | 0.513 | 0.727 | High | Cytosol, nucleoplasm, etc. |
| 162 | FMO5 | 0.745 | 0.963 | 0.519 | 1.011 | High | Cytosol, nucleoplasm, etc. |
| 163 | PPA1 | 0.745 | 0.816 | 0.597 | 0.680 | High | Secreted or membrane-bound |
| 164 | CTNNB1 | 0.744 | 0.682 | 0.728 | 0.546 | High | Secreted or membrane-bound |
| 165 | OCLN | 0.744 | 0.952 | 0.532 | 0.625 | High | Secreted or membrane-bound |
| 166 | C3 | 0.744 | 0.847 | 0.624 | 0.852 | High | Cytosol, nucleoplasm, etc. |
| 167 | AKAP13 | 0.743 | 0.815 | 0.593 | 0.579 | High | Cytosol, nucleoplasm, etc. |
| 168 | TKT | 0.743 | 0.624 | 0.787 | 0.565 | High | Cytosol, nucleoplasm, etc. |
| 169 | ST3GAL5 | 0.742 | 0.898 | 0.571 | 0.596 | High | Secreted or membrane-bound |
| 170 | NGFRAP1 | 0.742 | 0.781 | 0.676 | 0.652 | High | Cytosol, nucleoplasm, etc. |
| 171 | SDR16C5 | 0.742 | 0.944 | 0.537 | 0.789 | High | Cytosol, nucleoplasm, etc. |
| 172 | PLXNB2 | 0.741 | 0.871 | 0.599 | 0.568 | High | Cytosol, nucleoplasm, etc. |
| 173 | NEDD9 | 0.741 | 0.845 | 0.597 | 0.767 | High | Cytosol, nucleoplasm, etc. |
| 174 | TSC22D1 | 0.741 | 0.752 | 0.701 | 0.554 | High | Cytosol, nucleoplasm, etc. |
| 175 | ERBB3 | 0.741 | 0.953 | 0.525 | 0.569 | High | Cytosol, nucleoplasm, etc. |
| 176 | SPINK5 | 0.740 | 0.962 | 0.512 | 1.346 | High | Secreted or membrane-bound |
| 177 | H1F0 | 0.739 | 0.879 | 0.583 | 0.641 | High | Cytosol, nucleoplasm, etc. |
| 178 | F11R | 0.739 | 0.903 | 0.561 | 0.514 | High | Cytosol, nucleoplasm, etc. |
| 179 | FLNA | 0.739 | 0.824 | 0.578 | 0.563 | High | Secreted or membrane-bound |
| 180 | ARHGAP5 | 0.738 | 0.896 | 0.561 | 0.582 | High | Cytosol, nucleoplasm, etc. |
| 181 | EHF | 0.738 | 0.958 | 0.516 | 0.667 | High | Cytosol, nucleoplasm, etc. |
| 182 | SERINC2 | 0.737 | 0.912 | 0.552 | 0.611 | High | Secreted or membrane-bound |
| 183 | SFTPA1 | 0.737 | 0.806 | 0.633 | 0.752 | High | Cytosol, nucleoplasm, etc. |
| 184 | TACC2 | 0.736 | 0.951 | 0.513 | 0.807 | High | Cytosol, nucleoplasm, etc. |
| 185 | S100A16 | 0.735 | 0.866 | 0.597 | 0.654 | High | Cytosol, nucleoplasm, etc. |
| 186 | SCNN1A | 0.735 | 0.950 | 0.519 | 0.590 | High | Cytosol, nucleoplasm, etc. |
| 187 | PTPRC | 0.735 | 0.512 | 0.947 | -1.543 | Low | Cytosol, nucleoplasm, etc. |
| 188 | AK1 | 0.734 | 0.848 | 0.606 | 0.537 | High | Cytosol, nucleoplasm, etc. |
| 189 | C11orf96 | 0.734 | 0.898 | 0.557 | 1.257 | High | Cytosol, nucleoplasm, etc. |
| 190 | LAMB3 | 0.734 | 0.949 | 0.510 | 0.706 | High | Secreted or membrane-bound |
| 191 | MVP | 0.733 | 0.755 | 0.657 | 0.538 | High | Cytosol, nucleoplasm, etc. |
| 192 | GMFG | 0.732 | 0.518 | 0.927 | -1.381 | Low | Cytosol, nucleoplasm, etc. |
| 193 | BAIAP2 | 0.732 | 0.898 | 0.551 | 0.621 | High | Secreted or membrane-bound |
| 194 | STEAP4 | 0.732 | 0.939 | 0.518 | 0.954 | High | Secreted or membrane-bound |
| 195 | CLIC6 | 0.731 | 0.967 | 0.491 | 0.623 | High | Cytosol, nucleoplasm, etc. |
| 196 | SPRY4 | 0.731 | 0.970 | 0.487 | 0.899 | High | Cytosol, nucleoplasm, etc. |
| 197 | NCOA7 | 0.731 | 0.818 | 0.607 | 0.634 | High | Cytosol, nucleoplasm, etc. |
| 198 | NET1 | 0.730 | 0.907 | 0.539 | 0.640 | High | Cytosol, nucleoplasm, etc. |
| 199 | PPDPF | 0.730 | 0.788 | 0.601 | 0.595 | High | Cytosol, nucleoplasm, etc. |
| 200 | RRAD | 0.730 | 0.925 | 0.525 | 0.887 | High | Secreted or membrane-bound |
| 201 | CPD | 0.730 | 0.886 | 0.545 | 0.746 | High | Cytosol, nucleoplasm, etc. |
| 202 | CD2AP | 0.729 | 0.901 | 0.540 | 0.514 | High | Secreted or membrane-bound |
| 203 | ANK3 | 0.729 | 0.950 | 0.500 | 0.705 | High | Secreted or membrane-bound |
| 204 | GDF15 | 0.729 | 0.929 | 0.519 | 0.967 | High | Cytosol, nucleoplasm, etc. |
| 205 | PCBD1 | 0.728 | 0.739 | 0.669 | 0.514 | High | Cytosol, nucleoplasm, etc. |
| 206 | AFF4 | 0.728 | 0.796 | 0.619 | 0.505 | High | Cytosol, nucleoplasm, etc. |
| 207 | SMIM14 | 0.728 | 0.793 | 0.619 | 0.591 | High | Cytosol, nucleoplasm, etc. |
| 208 | GADD45G | 0.727 | 0.806 | 0.620 | 0.580 | High | Cytosol, nucleoplasm, etc. |
| 209 | IRX3 | 0.727 | 0.947 | 0.507 | 0.516 | High | Secreted or membrane-bound |
| 210 | STARD10 | 0.727 | 0.912 | 0.533 | 0.510 | High | Cytosol, nucleoplasm, etc. |
| 211 | HCST | 0.726 | 0.517 | 0.906 | -1.577 | Low | Cytosol, nucleoplasm, etc. |
| 212 | TFPI | 0.726 | 0.863 | 0.583 | 0.866 | High | Cytosol, nucleoplasm, etc. |
| 213 | ITGB6 | 0.726 | 0.942 | 0.510 | 0.664 | High | Cytosol, nucleoplasm, etc. |
| 214 | TMEM238 | 0.725 | 0.963 | 0.481 | 0.593 | High | Cytosol, nucleoplasm, etc. |
| 215 | SPINK13 | 0.725 | 0.992 | 0.456 | 1.010 | High | Secreted or membrane-bound |
| 216 | TSPAN13 | 0.725 | 0.892 | 0.553 | 0.528 | High | Cytosol, nucleoplasm, etc. |
| 217 | LIMCH1 | 0.725 | 0.914 | 0.535 | 0.504 | High | Cytosol, nucleoplasm, etc. |
| 218 | ELL2 | 0.723 | 0.808 | 0.595 | 0.669 | High | Cytosol, nucleoplasm, etc. |
| 219 | GADD45B | 0.723 | 0.765 | 0.595 | 0.877 | High | Cytosol, nucleoplasm, etc. |
| 220 | ANKRD36C | 0.723 | 0.916 | 0.503 | 1.642 | High | Cytosol, nucleoplasm, etc. |
| 221 | PLPP2 | 0.723 | 0.949 | 0.493 | 0.636 | High | Cytosol, nucleoplasm, etc. |
| 222 | PDZK1IP1 | 0.723 | 0.945 | 0.500 | 0.648 | High | Cytosol, nucleoplasm, etc. |
| 223 | LRRK2 | 0.722 | 0.918 | 0.518 | 0.905 | High | Secreted or membrane-bound |
| 224 | PURA | 0.722 | 0.865 | 0.553 | 0.542 | High | Cytosol, nucleoplasm, etc. |
| 225 | CD37 | 0.721 | 0.486 | 0.941 | -1.333 | Low | Cytosol, nucleoplasm, etc. |
| 226 | CXCL2 | 0.721 | 0.739 | 0.659 | 0.718 | High | Cytosol, nucleoplasm, etc. |
| 227 | CLINT1 | 0.721 | 0.810 | 0.590 | 0.526 | High | Secreted or membrane-bound |
| 228 | ITPR3 | 0.720 | 0.941 | 0.496 | 0.503 | High | Secreted or membrane-bound |
| 229 | MET | 0.720 | 0.964 | 0.473 | 0.676 | High | Secreted or membrane-bound |
| 230 | DUSP1 | 0.719 | 0.726 | 0.616 | 0.514 | High | Cytosol, nucleoplasm, etc. |
| 231 | MDK | 0.718 | 0.852 | 0.562 | 0.912 | High | Secreted or membrane-bound |
| 232 | CD53 | 0.718 | 0.479 | 0.944 | -1.312 | Low | Cytosol, nucleoplasm, etc. |
| 233 | F3 | 0.716 | 0.925 | 0.501 | 0.850 | High | Secreted or membrane-bound |
| 234 | ANGPTL4 | 0.715 | 0.955 | 0.469 | 1.053 | High | Secreted or membrane-bound |
| 235 | SELENBP1 | 0.714 | 0.897 | 0.532 | 0.518 | High | Cytosol, nucleoplasm, etc. |
| 236 | CYP4B1 | 0.713 | 0.940 | 0.478 | 0.727 | High | Secreted or membrane-bound |
| 237 | AZGP1 | 0.711 | 0.964 | 0.455 | 0.864 | High | Cytosol, nucleoplasm, etc. |
| 238 | SLC25A37 | 0.711 | 0.832 | 0.557 | 0.600 | High | Cytosol, nucleoplasm, etc. |
| 239 | CTSE | 0.710 | 0.961 | 0.455 | 0.885 | High | Cytosol, nucleoplasm, etc. |
| 240 | FCHO2 | 0.709 | 0.903 | 0.506 | 0.554 | High | Cytosol, nucleoplasm, etc. |
| 241 | OSMR | 0.709 | 0.939 | 0.476 | 0.507 | High | Cytosol, nucleoplasm, etc. |
| 242 | SESTD1 | 0.708 | 0.912 | 0.493 | 0.630 | High | Cytosol, nucleoplasm, etc. |
| 243 | MIR4458HG | 0.707 | 0.917 | 0.487 | 0.580 | High | Cytosol, nucleoplasm, etc. |
| 244 | SERPINA1 | 0.707 | 0.667 | 0.728 | 0.560 | High | Secreted or membrane-bound |
| 245 | SEZ6L2 | 0.707 | 0.964 | 0.448 | 0.519 | High | Cytosol, nucleoplasm, etc. |
| 246 | TRIB1 | 0.702 | 0.872 | 0.513 | 0.543 | High | Secreted or membrane-bound |
| 247 | FASN | 0.701 | 0.924 | 0.475 | 0.679 | High | Secreted or membrane-bound |
| 248 | HIST1H1C | 0.701 | 0.857 | 0.521 | 0.508 | High | Cytosol, nucleoplasm, etc. |
| 249 | AGRN | 0.700 | 0.927 | 0.466 | 0.556 | High | Secreted or membrane-bound |
| 250 | SNX30 | 0.699 | 0.935 | 0.457 | 0.522 | High | Cytosol, nucleoplasm, etc. |
| 251 | CRABP2 | 0.698 | 0.937 | 0.453 | 0.757 | High | Cytosol, nucleoplasm, etc. |
| 252 | NDRG1 | 0.696 | 0.840 | 0.526 | 0.653 | High | Cytosol, nucleoplasm, etc. |
| 253 | WEE1 | 0.696 | 0.933 | 0.454 | 0.514 | High | Cytosol, nucleoplasm, etc. |
| 254 | ALCAM | 0.694 | 0.822 | 0.549 | 0.558 | High | Cytosol, nucleoplasm, etc. |
| 255 | CORO1A | 0.694 | 0.457 | 0.910 | -1.474 | Low | Cytosol, nucleoplasm, etc. |
| 256 | RNF213 | 0.694 | 0.868 | 0.450 | 0.522 | High | Cytosol, nucleoplasm, etc. |
| 257 | IGFBP2 | 0.693 | 0.882 | 0.489 | 1.048 | High | Cytosol, nucleoplasm, etc. |
| 258 | MUC4 | 0.693 | 0.980 | 0.402 | 0.902 | High | Cytosol, nucleoplasm, etc. |
| 259 | IL1R1 | 0.691 | 0.910 | 0.465 | 0.586 | High | Secreted or membrane-bound |
| 260 | HPGD | 0.691 | 0.878 | 0.481 | 0.966 | High | Cytosol, nucleoplasm, etc. |
| 261 | ALOX15B | 0.689 | 0.959 | 0.416 | 0.518 | High | Secreted or membrane-bound |
| 262 | KLF6 | 0.687 | 0.874 | 0.452 | 0.634 | High | Cytosol, nucleoplasm, etc. |
| 263 | LSP1 | 0.684 | 0.420 | 0.935 | -1.205 | Low | Cytosol, nucleoplasm, etc. |
| 264 | ALOX5AP | 0.684 | 0.471 | 0.855 | -1.635 | Low | Cytosol, nucleoplasm, etc. |
| 265 | LDLR | 0.683 | 0.889 | 0.465 | 0.536 | High | Cytosol, nucleoplasm, etc. |
| 266 | SLC5A3 | 0.681 | 0.875 | 0.474 | 0.541 | High | Secreted or membrane-bound |
| 267 | TNC | 0.680 | 0.964 | 0.396 | 0.550 | High | Cytosol, nucleoplasm, etc. |
| 268 | ID1 | 0.680 | 0.819 | 0.530 | 0.575 | High | Cytosol, nucleoplasm, etc. |
| 269 | GGTLC1 | 0.679 | 0.963 | 0.393 | 0.549 | High | Cytosol, nucleoplasm, etc. |
| 270 | CCL4 | 0.679 | 0.353 | 0.979 | -2.646 | Low | Cytosol, nucleoplasm, etc. |
| 271 | LCP1 | 0.677 | 0.403 | 0.942 | -1.067 | Low | Cytosol, nucleoplasm, etc. |
| 272 | SOX9 | 0.677 | 0.973 | 0.379 | 0.522 | High | Cytosol, nucleoplasm, etc. |
| 273 | RGMB | 0.677 | 0.976 | 0.375 | 0.529 | High | Cytosol, nucleoplasm, etc. |
| 274 | CD24 | 0.676 | 0.917 | 0.429 | 0.685 | High | Secreted or membrane-bound |
| 275 | SFTA1P | 0.676 | 0.937 | 0.412 | 0.574 | High | Cytosol, nucleoplasm, etc. |
| 276 | TNFSF10 | 0.675 | 0.795 | 0.528 | 0.600 | High | Cytosol, nucleoplasm, etc. |
| 277 | CEACAM5 | 0.674 | 0.980 | 0.367 | 0.860 | High | Secreted or membrane-bound |
| 278 | CYTIP | 0.674 | 0.386 | 0.956 | -1.172 | Low | Cytosol, nucleoplasm, etc. |
| 279 | RGS1 | 0.673 | 0.384 | 0.959 | -2.010 | Low | Cytosol, nucleoplasm, etc. |
| 280 | BTG1 | 0.672 | 0.523 | 0.761 | -1.757 | Low | Cytosol, nucleoplasm, etc. |
| 281 | MUC21 | 0.671 | 0.984 | 0.355 | 0.663 | High | Cytosol, nucleoplasm, etc. |
| 282 | ID2 | 0.671 | 0.468 | 0.851 | -1.478 | Low | Cytosol, nucleoplasm, etc. |
| 283 | FCER1G | 0.669 | 0.372 | 0.974 | -1.907 | Low | Cytosol, nucleoplasm, etc. |
| 284 | MACC1 | 0.668 | 0.949 | 0.381 | 0.560 | High | Cytosol, nucleoplasm, etc. |
| 285 | TYROBP | 0.667 | 0.405 | 0.947 | -2.199 | Low | Cytosol, nucleoplasm, etc. |
| 286 | NQO1 | 0.666 | 0.927 | 0.404 | 0.535 | High | Cytosol, nucleoplasm, etc. |
| 287 | SCGB3A2 | 0.665 | 0.762 | 0.525 | 1.089 | High | Cytosol, nucleoplasm, etc. |
| 288 | VSTM2L | 0.665 | 0.980 | 0.349 | 0.552 | High | Cytosol, nucleoplasm, etc. |
| 289 | CD69 | 0.664 | 0.381 | 0.934 | -1.742 | Low | Cytosol, nucleoplasm, etc. |
| 290 | SUSD2 | 0.664 | 0.939 | 0.381 | 0.925 | High | Secreted or membrane-bound |
| 291 | ZNF385B | 0.663 | 0.966 | 0.359 | 0.625 | High | Cytosol, nucleoplasm, etc. |
| 292 | CCL5 | 0.663 | 0.375 | 0.938 | -2.375 | Low | Cytosol, nucleoplasm, etc. |
| 293 | PHACTR3 | 0.662 | 0.985 | 0.336 | 1.247 | High | Cytosol, nucleoplasm, etc. |
| 294 | LCN2 | 0.661 | 0.952 | 0.369 | 0.520 | High | Cytosol, nucleoplasm, etc. |
| 295 | CDKN1C | 0.660 | 0.912 | 0.402 | 0.574 | High | Cytosol, nucleoplasm, etc. |
| 296 | GYPC | 0.660 | 0.348 | 0.966 | -0.898 | Low | Cytosol, nucleoplasm, etc. |
| 297 | SAMSN1 | 0.659 | 0.358 | 0.952 | -1.136 | Low | Cytosol, nucleoplasm, etc. |
| 298 | RAC2 | 0.658 | 0.418 | 0.871 | -1.181 | Low | Cytosol, nucleoplasm, etc. |
| 299 | IRS2 | 0.657 | 0.832 | 0.457 | 0.520 | High | Cytosol, nucleoplasm, etc. |
| 300 | B2M | 0.656 | 0.574 | 0.672 | -1.610 | Low | Cytosol, nucleoplasm, etc. |
| 301 | EDN1 | 0.656 | 0.962 | 0.346 | 0.541 | High | Cytosol, nucleoplasm, etc. |
| 302 | TNNC2 | 0.655 | 0.984 | 0.323 | 0.927 | High | Cytosol, nucleoplasm, etc. |
| 303 | LINC00342 | 0.654 | 0.981 | 0.324 | 0.624 | High | Cytosol, nucleoplasm, etc. |
| 304 | CD48 | 0.651 | 0.322 | 0.978 | -1.015 | Low | Cytosol, nucleoplasm, etc. |
| 305 | EVI2B | 0.649 | 0.325 | 0.966 | -0.871 | Low | Cytosol, nucleoplasm, etc. |
| 306 | FYB | 0.648 | 0.325 | 0.968 | -0.930 | Low | Cytosol, nucleoplasm, etc. |
| 307 | ITGA2 | 0.648 | 0.969 | 0.324 | 0.508 | High | Cytosol, nucleoplasm, etc. |
| 308 | EMP3 | 0.647 | 0.447 | 0.825 | -1.273 | Low | Cytosol, nucleoplasm, etc. |
| 309 | PTPN13 | 0.647 | 0.948 | 0.343 | 0.523 | High | Cytosol, nucleoplasm, etc. |
| 310 | IL2RG | 0.647 | 0.334 | 0.957 | -1.017 | Low | Cytosol, nucleoplasm, etc. |
| 311 | TFF3 | 0.645 | 0.952 | 0.335 | 0.898 | High | Cytosol, nucleoplasm, etc. |
| 312 | AQP5 | 0.645 | 0.970 | 0.317 | 0.754 | High | Secreted or membrane-bound |
| 313 | SH3BGRL3 | 0.644 | 0.601 | 0.634 | -1.423 | Low | Cytosol, nucleoplasm, etc. |
| 314 | COTL1 | 0.640 | 0.336 | 0.928 | -1.171 | Low | Cytosol, nucleoplasm, etc. |
| 315 | TRAC | 0.639 | 0.317 | 0.953 | -1.590 | Low | Cytosol, nucleoplasm, etc. |
| 316 | HSPA6 | 0.639 | 0.850 | 0.417 | 0.584 | High | Secreted or membrane-bound |
| 317 | CD2 | 0.638 | 0.303 | 0.969 | -1.397 | Low | Cytosol, nucleoplasm, etc. |
| 318 | NKG7 | 0.636 | 0.303 | 0.962 | -2.257 | Low | Cytosol, nucleoplasm, etc. |
| 319 | SPINK1 | 0.636 | 0.969 | 0.299 | 1.418 | High | Cytosol, nucleoplasm, etc. |
| 320 | CD3D | 0.635 | 0.320 | 0.941 | -1.520 | Low | Cytosol, nucleoplasm, etc. |
| 321 | FKBP5 | 0.634 | 0.940 | 0.305 | 0.558 | High | Cytosol, nucleoplasm, etc. |
| 322 | C1orf162 | 0.633 | 0.299 | 0.957 | -0.989 | Low | Cytosol, nucleoplasm, etc. |
| 323 | DOK2 | 0.631 | 0.274 | 0.985 | -0.743 | Low | Cytosol, nucleoplasm, etc. |
| 324 | XIST | 0.630 | 0.919 | 0.364 | 0.593 | High | Cytosol, nucleoplasm, etc. |
| 325 | ZEB2 | 0.630 | 0.280 | 0.977 | -0.744 | Low | Cytosol, nucleoplasm, etc. |
| 326 | GLIPR2 | 0.627 | 0.284 | 0.965 | -0.689 | Low | Cytosol, nucleoplasm, etc. |
| 327 | SFTPD | 0.626 | 0.908 | 0.343 | 0.767 | High | Cytosol, nucleoplasm, etc. |
| 328 | TRBC2 | 0.626 | 0.300 | 0.944 | -1.352 | Low | Cytosol, nucleoplasm, etc. |
| 329 | IL7R | 0.625 | 0.283 | 0.964 | -1.283 | Low | Cytosol, nucleoplasm, etc. |
| 330 | MAP1B | 0.625 | 0.943 | 0.304 | 0.547 | High | Cytosol, nucleoplasm, etc. |
| 331 | HCLS1 | 0.625 | 0.289 | 0.954 | -0.707 | Low | Cytosol, nucleoplasm, etc. |
| 332 | SLA | 0.625 | 0.274 | 0.973 | -0.839 | Low | Cytosol, nucleoplasm, etc. |
| 333 | GZMA | 0.624 | 0.269 | 0.976 | -1.630 | Low | Cytosol, nucleoplasm, etc. |
| 334 | GLIPR1 | 0.624 | 0.302 | 0.938 | -0.723 | Low | Cytosol, nucleoplasm, etc. |
| 335 | CREM | 0.623 | 0.331 | 0.933 | -1.429 | Low | Cytosol, nucleoplasm, etc. |
| 336 | WIPF1 | 0.623 | 0.299 | 0.940 | -0.724 | Low | Cytosol, nucleoplasm, etc. |
| 337 | PGC | 0.622 | 0.930 | 0.311 | 0.624 | High | Cytosol, nucleoplasm, etc. |
| 338 | CD3E | 0.620 | 0.263 | 0.976 | -1.114 | Low | Cytosol, nucleoplasm, etc. |
| 339 | LST1 | 0.620 | 0.239 | 0.993 | -1.152 | Low | Cytosol, nucleoplasm, etc. |
| 340 | SLC2A3 | 0.618 | 0.339 | 0.886 | -0.885 | Low | Cytosol, nucleoplasm, etc. |
| 341 | CST7 | 0.618 | 0.296 | 0.936 | -1.246 | Low | Cytosol, nucleoplasm, etc. |
| 342 | ITGB2 | 0.618 | 0.302 | 0.915 | -1.034 | Low | Cytosol, nucleoplasm, etc. |
| 343 | FCGR3A | 0.614 | 0.260 | 0.959 | -1.039 | Low | Cytosol, nucleoplasm, etc. |
| 344 | CD99 | 0.614 | 0.393 | 0.831 | -0.974 | Low | Cytosol, nucleoplasm, etc. |
| 345 | RHOG | 0.612 | 0.360 | 0.837 | -0.681 | Low | Cytosol, nucleoplasm, etc. |
| 346 | DUSP2 | 0.612 | 0.288 | 0.937 | -1.402 | Low | Cytosol, nucleoplasm, etc. |
| 347 | AIF1 | 0.612 | 0.261 | 0.986 | -1.412 | Low | Cytosol, nucleoplasm, etc. |
| 348 | GPSM3 | 0.611 | 0.256 | 0.940 | -0.849 | Low | Cytosol, nucleoplasm, etc. |
| 349 | CD7 | 0.611 | 0.277 | 0.936 | -1.294 | Low | Cytosol, nucleoplasm, etc. |
| 350 | AQP1 | 0.611 | 0.894 | 0.320 | 0.880 | High | Secreted or membrane-bound |
| 351 | MUC5B | 0.610 | 0.988 | 0.233 | 0.687 | High | Secreted or membrane-bound |
| 352 | EVI2A | 0.607 | 0.223 | 0.991 | -0.615 | Low | Cytosol, nucleoplasm, etc. |
| 353 | CCL3 | 0.607 | 0.225 | 0.987 | -1.988 | Low | Cytosol, nucleoplasm, etc. |
| 354 | GIMAP4 | 0.607 | 0.229 | 0.982 | -0.710 | Low | Cytosol, nucleoplasm, etc. |
| 355 | SPI1 | 0.605 | 0.240 | 0.965 | -0.785 | Low | Cytosol, nucleoplasm, etc. |
| 356 | CYBA | 0.605 | 0.651 | 0.563 | -1.090 | Low | Cytosol, nucleoplasm, etc. |
| 357 | TRBC1 | 0.605 | 0.239 | 0.965 | -1.323 | Low | Cytosol, nucleoplasm, etc. |
| 358 | FXYD5 | 0.605 | 0.422 | 0.778 | -0.909 | Low | Cytosol, nucleoplasm, etc. |
| 359 | INHBB | 0.604 | 0.982 | 0.223 | 0.518 | High | Cytosol, nucleoplasm, etc. |
| 360 | STK4 | 0.603 | 0.364 | 0.816 | -0.941 | Low | Cytosol, nucleoplasm, etc. |
| 361 | CTSW | 0.603 | 0.223 | 0.980 | -1.132 | Low | Cytosol, nucleoplasm, etc. |
| 362 | S100P | 0.602 | 0.958 | 0.244 | 0.598 | High | Cytosol, nucleoplasm, etc. |
| 363 | BCL2A1 | 0.602 | 0.230 | 0.969 | -0.922 | Low | Cytosol, nucleoplasm, etc. |
| 364 | GPR65 | 0.602 | 0.219 | 0.983 | -0.728 | Low | Cytosol, nucleoplasm, etc. |
| 365 | PPP1R18 | 0.602 | 0.279 | 0.916 | -0.618 | Low | Cytosol, nucleoplasm, etc. |
| 366 | RGCC | 0.601 | 0.337 | 0.871 | -1.251 | Low | Cytosol, nucleoplasm, etc. |
| 367 | ECM1 | 0.601 | 0.942 | 0.255 | 0.765 | High | Cytosol, nucleoplasm, etc. |
| 368 | GZMB | 0.601 | 0.232 | 0.964 | -1.802 | Low | Cytosol, nucleoplasm, etc. |
| 369 | S100A8 | 0.600 | 0.242 | 0.951 | -2.052 | Low | Cytosol, nucleoplasm, etc. |
